# Attitudes to climate change risk: classification of and transitions in the UK population between 2012 and 2020

**DOI:** 10.1057/s41599-022-01287-1

**Published:** 2022-08-18

**Authors:** Ting Liu, Nick Shryane, Mark Elliot

**Affiliations:** The Cathie Marsh Institute for Social Research, School of Social Sciences, University of Manchester, Manchester, UK

**Keywords:** Environmental studies, Psychology, Sociology

## Abstract

Strategies for achieving carbon emissions goals presuppose changes in individual behaviour, which can be indirectly nudged by interventions or tailored information but ultimately depend upon individual attitudes. Specifically, the perception that climate change is low risk has been identified as a barrier to participation in climate change adaptation and mitigation efforts. Therefore, understanding public attitudes towards climate change risk is an important element of reducing emissions. We applied *k*-means cluster analysis to explore attitudes to climate change risk in the UK population using data from the UK Household Longitudinal Study, a national survey running from 2009 to present. We identified three distinct attitude clusters: “Sceptical”, “Concerned”, and “Paradoxical” in both waves 4 (from 2012 to 2014) and 10 (from 2018 to 2020) of this survey. The Sceptical cluster tended to deny the seriousness of climate change and the urgency or even the necessity of dealing with it. The Concerned cluster displayed anxiety about climate change risks and supported action to reduce them. The Paradoxical cluster acknowledged the reality of climate change impacts but did not support actions to mitigate them. We further observed statistical associations between cluster membership and the social characteristics of the participants, including sex, age, income, education, and political affiliation. We also found a temporal stability of cluster structure between the two waves. However, the transition matrices indicated a general transition away from the Sceptical and Paradoxical clusters, and toward the Concerned cluster between wave 4 to wave 10. The findings suggest that more tailored public information campaigns regarding climate change risk may be necessary.

## Introduction

In order to play its part in meeting the 1.5 degree Celsius global warming target—set in the Paris Climate Agreement—the UK has a long-term goal to achieve net-zero emissions by 2050 (Committe on Climate Change, 2019; United Nations, 2015). As well as requiring institutional changes in policy, individual behaviour changes will be needed to achieve this goal. This, in turn, may be dependent on the public’s beliefs about the seriousness of climate change and the urgency of action to mitigate it.

Analysis of UK national survey data has presented a complex picture of public perceptions of the risk of climate change. On the one hand, many British people believe climate change is serious. For instance, respondents’ support for the statement that “the seriousness of climate change is exaggerated” was 40% in 2010 to 30% in 2012 and 34% in 2013 (Poortinga et al., 2014). Similarly, a 2015 Pew Research Center (2015) poll found that 77% of people in the UK recognise global climate change as a very or somewhat serious problem. On the other hand, some people are in denial about the existence of climate change. Poortinga et al. (2011) found that 28% of people were uncertain about the existence of climate change. However, a more recent study conducted in 2018 indicated that complete scepticism about climate change is now unusual; with only 6% of the respondents being classed as deniers and many of those are not overly confident in their beliefs (Crawley et al., 2020).

The foregoing raises the questions of how attitudes toward and beliefs about climate change risk are organised and how they vary. As many authors have pointed out, the perception that climate change is low risk constitutes a barrier to participation in climate change adaptation and mitigation efforts (see, for example, Spence et al., 2011; Weber, 2011). However, merely describing public attitudes to climate change based on single-item measuring people’s agreement on seriousness of climate change might constrain our understanding of how people view its risk and how risk attitudes in different population segments might vary in nuanced ways. Previous work has provided snapshots of the heterogeneity of general assessments of climate change and/or wider environmental issues; they have focused on understanding people’s concern about climate change using a broad set of items relating to values, beliefs, willingness to support mitigation policy or behaviour. Consequently, they have not specifically focused on how people view the risks of climate or environmental change and understanding that specific element is critical in thinking constructively about behaviour change. In addition, most environmental and climate change segmentation studies in the UK have employed cross-sectional data and leaving open questions about change over time. As public attitudes to climate change have changed, it is, as Detenber and Rosenthal (2020) observe, valuable to monitor the composition and trajectory of population clusters over time. The current study explores the clustering of attitudes to climate change risk (ACCRs) that existed in the UK during the progress toward the GHG emissions reduction targets, and how the population segmentation of ACCRs both varies by socio-demographics and has changed over time. The findings will assist practitioners in the construction of communication frames; informing the tailoring and targeting of messages for members of each cluster, to more effectively maintain and/or enhance climate change engagement in each cluster.

The remainder of this paper is organised as follows. Section “Background” reviews relevant literature and develops research questions. Sections “The current study” and “Methods” outlines the methods employed, section “Results” presents the results and section “Discussion” discusses implications of the research, its limitations and possibilities for future research.

## Background

### Attitudes to climate change risk

Attitudes to Climate Change Risk (ACCRs) are generally considered to be consonant with the companion concept of *climate change risk perception*; and the two terms are often used interchangeably in the climate change research literature. Risk perception is a subjective construct often conceptualised as the perception of potential damage or harm (Slovic, 2000). To measure climate change risk perception, some researchers have utilised a variety of intermediate constructs including: perceived seriousness (McCright et al., 2014b), general concern about climate change threats (Milfont, 2012), likelihood measures of the various impact of global and/or local climate change will have in the future (Akerlof et al., 2013; Brody et al., 2008), the extent and timing of harms (Ding et al., 2011), while others adopt a combination of all or some of these measures (e.g., Leiserowitz, 2006; van der Linden, 2015).

In this study, we follow van der Linden (2017) in using ACCRs to refer to the combination of the perception of the probability that climate change will happen and the affective response to the perceived severity of negative climate-related consequences. This definition reflects a bipartite theoretical position. On the one hand, the cognitive process underlying ACCRs is based on knowledge about climate change, which includes the existence, cause, impacts and solutions of climate change (Tobler et al., 2012; van der Linden, 2015). On the other hand, the affective processing of climate change involves apprehension and worry about the potential negative impacts of climate change (Arbuckle et al., 2013).

In considering climate risk, people may distinguish between personal and societal implications (Bord et al., 1998), or they may perceive this as a risk for future generations, or as something out of their personal control (Poortinga and Pidgeon, 2003). The temporal perception of climate change is a critical component of the cognitive element of ACCRs and concerns when its effects are felt and who will feel them (e.g., Poortinga et al., 2011). Spatial perception is also important and may produce biases based on self-other comparisons (i.e., if the individual perceives that they are less likely to experience the effects of climate change than others are; Pahl et al., 2005) and geographic distance (i.e., local conditions are better than global conditions, Gifford et al., 2009). Hence, people do not always view climate change as personally threatening or relevant to them. van der Linden (2017) developed a “hierarchy of concern” model, which hypothesised climate change risk perception might be represented by a transitive relationship: “An individual may think that climate change (and associated impacts) are likely to occur, but that doesn’t mean that someone also perceives climate change to be a serious issue. In turn, an individual can perceive climate change to be a serious issue, but that doesn’t necessarily imply that they are concerned about it. Finally, although the public may express generalised concern about climate change, this often does not mean that people also personally worry about the issue or think it is a high priority.” (p. 24).

### Population segmentation of climate change attitudes

Climate change is slow-moving, cumulative, and unsituated (Van Vugt et al., 2014). Consequently, people do not experience the change directly or even detect it on a sensory level. This is accentuated by the global scale and abstract statistical nature of our climate; covering as it does long-term changes of the whole of the world, with varying effects in particular localities (Weber, 2010). Public perceptions are therefore heterogeneous with risk attitudes being driven by extraneous social and psychological characteristics (Sciullo et al., 2019; van der Linden, 2017).

Several recent studies have sought to organise people’s climate change beliefs and attitudes based on homogenous item response patterns (see, for example, Maibach et al., 2011; Myers et al., 2012; Rhead et al., 2018; Sibley and Kurz, 2013). Using a survey of 2164 respondents in the US, with 36 ordinal and nominal variables related to beliefs in global warming, degree of involvement in the issue, behaviours, and policy preference, Maibach et al. (2011) used Latent Class Analysis (LCA) to classify six audience segments: Alarmed, Concerned, Cautious, Disengaged, Doubtful, and Dismissive, which they refer to as the *Six Americas*. Subsequently, Myers et al. (2012) tested Maibach et al.’s segments of US residents (*N* = 1127) and compared the effectiveness of climate change message framings among the segments. Conversely, Sibley and Kurz (2013) used data from New Zealand and produced a four-class model consisting of Climate Believers, Undecided/Neutral, Climate Sceptics, and Anthropogenic Climate Sceptics. Narrower classes also have been found in the UK nationally representative survey; Rhead et al. (2018) produced four classes of concern for the environment (including climate change): Pro-environment, Neutral Majority, Disengaged, and Paradoxical. Most recently, Crawley et al. (2020) used LCA to segment five climate change opinion groups (Highly Engaged, Moderately Engaged, Action-wary, Uncertain, and Deniers) in 2018 in the UK (*N* = 787).

Other studies have used cluster analysis to segment the population into broader environmental attitude groups. For example, DEFRA (2008) used cluster analysis and contextualised interviews to produce a seven-category UK-wide environmental typology ranging from Positive Greens to Honestly Disengaged. Thornton et al. (2011) identified nine clusters of attitudes to transport choices based on the interviews conducted with English residents. Their segments combined attitudes and behaviour patterns with socioeconomic groups, with divisions ranging from Older, less mobile car owners to Young urbanites without cars. Waitt et al. (2012) clustered climate change responses into three segments: Strong Engagement, Modest Engagement, and Limited Engagement using Ward’s and *k*-means cluster analysis in a single Australian city (Wollongong). In Australia, Ashworth et al. (2011) identified a four-category segmentation ranging from Engaged to Doubtful using a two-stage cluster analysis (unspecified hierarchical and *k*-means). Similar segmentations also produced in a multi-national sample, a recent work conducted by Kácha et al. (2022) classified four groups of climate change beliefs and attitudes in 23 countries in Europe: Engaged, Pessimistic, Indifferent, and Doubtful.

Beyond the cross-sectional analysis of population classifications, some researchers have attempted to track changes of climate change values, beliefs, or behaviours clusters over time. For example, Leiserowitz et al. (2011) explored the changes in segment size of the so-called “Six Americas” from 2008 through 2011. However, Leiserowitz and colleagues assumed a fixed and constant cluster solution across time and specifically that the original Six Americas cluster structure identified in the 2008 data remain stable over time, which ignores the possibility that segment structure may change across time or that previous clusters might combine or split (Hine et al., 2014). Conversely, Heberlein (2012) provides examples where environmental attitude clusters do appear to disappear or merge into other clusters as people age and/or experience period effects (such as major environmental disasters or extreme weathers). Nevertheless, such changes in attitudes take time and do not necessarily lead to changes in overall attitudinal structures.

It follows from the above, that it is reasonable to track population shifts between segments in ways that are both sensitive to the emergence of new cluster structures and driven by the actual data structure, rather than constrained by the solutions from previous studies. To date, there are still gaps in our knowledge of the trajectory of attitudes toward climate change risks across time. Therefore, there is a need to draw a picture of attitudes transition adopting approaches that may produce more precise and valid change measures.

A review of past work on the climate change attitude population segmentation suggests that LCA and cluster analysis are two popular approaches. Given its ability to analyse both categorical and interval-scaled variables, include cases with missing data, and access model fit (McLachlan and Peel, 2000), LCA has been the preferred strategy for generating such segmentation (Hine et al., 2014). However, for segmenting large national survey datasets (especially for the sample sizes over ten thousand), its efficiency might be limited by the potentially large numbers of clusters. For instance, small and idiosyncratic response patterns can contribute to the model fit when LCA is coupled with very large sample size, resulting in a large number of classes that are hard to interpret substantively and a risk that mispecified models are absorbed into spurious latent classes (Bauer and Curran, 2004). LCA is also more likely to encounter convergence problems, while cluster analysis methods (such as *K*-mean clustering) are more likely to converge with larger samples and in general will be computationally robust as the sample size increases (see Vermunt, 2011).

So, following the above reasoning, this study will generate ACCRs group using *k*-means cluster analysis in a national survey dataset (with a sample size over ten thousand) and will use as the basis for an analysis of cross-time cluster transitions (at the individual and population levels).

### Socio-demographic characteristics and climate change risk attitudes

An important area of study has been the exploration of how socio-demographic variables (e.g., education, age, gender, income, and political affiliation) predict climate change and environmental attitudes. Evidence on the influence of such socio-demographic factors is mixed with results varying from sample to sample and study to study.

#### (Binary) Sex

Some studies have found that males are more likely to be deniers of climate change than females (Clements, 2012; McCright, 2010). Milfont and Sibley (2016) theorised that women tend to be socialised to have higher levels of empathy than men, leading them to hold stronger pro-environmental attitudes and heightened perception of the risk of environmental impoverishment. However, other studies have found that females were less environmentally concerned than males. McEvoy (1972) attributed this to men having higher levels of education (at the time) and greater involvement in the community and political issues. Yet others found no significant effect of gender on environmental concern (e.g., Melis et al., 2014).

#### Age

Results are equally inconsistent for age, with some studies revealing a small negative correlation between age and climate change perception, (e.g., see Heath and Gifford, 2006; Hornsey et al., 2016), while others find no significant effect (e.g., Akerlof et al., 2013; O’Connor et al., 1999) or a positive correlation (Slimak and Dietz, 2006). Most of the studies suggested that age was inversely related to more general environmental attitudes, with younger cohorts tending to be more environmentally concerned than older cohorts (e.g., see Gökşen et al., 2002; Rhead et al., 2018).

#### Income

On average, people with higher income report stronger beliefs of climate change—yet lower risk perception—compared to those with less income (Xiao and McCright, 2012). One of the explanations for this is that income is a proxy measure of social class/social grade, partially because it shapes the social contexts where people are exposed to, and share information (Kraus and Stephens, 2012) and partly because people of higher social class and economic position are more likely to be risk-averse given that environmental challenges represent a risk to the hegemony of industrial capitalism and with it their social position (Ballew et al., 2020; McCright, 2011). Similarly, Rhead et al. (2018) found that Britain’s highest social grade (managerial/professional) had the highest proportion of pro-environmental attitudes and behaviours. For those who might have lower income, Rhead et al. (2018) found that non-workers, pensioners, or job-seekers are inclined to moderately regard climate change as a low priority and hold apathetic attitudes towards environmental issues despite acknowledging the risks.

#### Political affiliation

Partisanship is envisaged as the largest demographic correlate of attitudes to climate change (Hornsey et al., 2016; Davidson and Haan, 2012). People who vote for left-leaning parties are more likely to view climate change as risky than those who support mainstream-right political parties (Zahran et al., 2006; Davidson and Haan, 2012). Within the US, the tendency for Republicans (right-wing) to express more scepticism than Democrats (left-wing) has long been observed and is credited with contributing to a growing ideological gulf between sceptics and non-sceptics (McCright and Dunlap, 2011b; McCright et al., 2013, 2014a). In the majority of European countries, Gregersen et al. (2020) found that self-positioning further to the right of the political spectrum is correlated with lower levels of worry about climate change. Melis et al. (2014) found that voting for the UK Conservative Party (right-wing) is associated with lower levels of environmental concern than any other voting choice (including non-voting). Similarly, research by Phillips et al. (2018) found that support for climate change mitigation policies is slightly higher for Labour voters (Mainstream-left) than for Conservative (Mainstream-right) voters. However, recent research conducted post-COVID-19 in the UK has shown that even Conservative voters prefer the environment to be at the heart of economic reconstruction (Kenward and Brick, 2021).

#### Education

Again, the results for level of education are mixed. On one hand, having attended higher education predicts stronger perception of the risk of climate change (e.g., Hornsey et al., 2016; Lee et al., 2015; van der Linden, 2015) and less denial about climate change (Boag et al., 2016). On the other hand, years of education has also been found to have an inverse relationship with concern about climate change (e.g., Malka et al., 2009; Slimak and Dietz, 2006). Other studies, for instance, Melis et al. (2014) and Rhead et al. (2018) found that education level is not a significant predictor for environmental concern in the UK population once other factors are controlled for. This may partly be because the effects of education on concern about climate change tend to interact with those of political affiliation (Hamilton, 2011). People tend to acknowledge and interpret information through the filter off their existing value and belief systems (Taber and Lodge, 2006) and are more inclined to develop and reinforce attitude systems based on the cultural worldviews and social groups to which they belong (Kahan et al., 2007). For example, groups that have a general tendency to dismiss climate change (e.g., white-male right-wingers) may, when exposed to politicised issues like climate change through say higher education, interpret information in a way that bolsters value and attitudes systems associated with groups of which they are members (Drummond and Fischhoff, 2017). Hence, the probability of perceiving climate change as risky increases with education among those with left-wing political affiliation but decreases with education among right-wingers.

In a nutshell, despite the inconsistent results, there is some evidence for a socio-demographic risk profile where typically younger, female, higher educated, those with higher income and a left-leaning political affiliation express more concern about climate change. Hence, here we will examine how population segments with different ACCRs vary by age, gender, income, education, and political affiliation.

## The current study

Although the majority of previous research has focused on the latent variable analysis of general concern about the environment or climate change, such as four environmental concern clusters partitioned in the British population by Rhead et al. (2018) and five climate change opinion groups in the UK by Crawley et al. (2020), to date little research has been focused on cluster analysis of ACCRs and cluster trajectory across time. The questions of how people perceive and are concerned about the timescale, trend, consequences, and crisis of climate change has been largely unstudied. Shifting from the focus on general concern to the focus on how people view risks of climate change specifically would enable communications and interventions to be tailored towards specific groups (with varying response patterns to climate change risk statements). The present study seeks to explore ACCRs using *k*-means cluster analysis in order to identify statistically distinct response clusters across time.

How environmental concern cluster membership varies by socio-demographics has been tested in previous studies, but the findings have been mixed. Additionally, the demographics of cluster membership may only be consistent with those found previously if our ACCRs clusters are themselves isomorphic with the environmental concern clusters extracted in previous studies. Therefore, we will examine whether the probability of belonging to any of the ACCRs clusters that we identify is influenced by individuals’ sex, age, income, education, and political affiliation without any pre-determined hypothesis.

The study uses the climate change module with the UK Household Longitudinal Study (UKHLS) from waves 4 and 10 (which were collected between 2012 and 2014, and 2018 and 2020, respectively).

The goal of the present study was therefore to answer three key research questions:

1. What groups of ACCRs exist in the UK?
2. What is the relationship between socio-demographic variables and ACCRs group membership?
3. How do ACCRs group structure and membership change over time?

## Methods

### Data and sample

Our analysis uses the UK Household Longitudinal Study (UKHLS)^1^; an annual panel survey of UK households. The first wave was conducted in 2009 included approximately 40,000 households and 100,000 individuals. These households were initially selected using a multistage random sampling (a random sample of addresses drawn from a random sample of UK post codes) and used multiple sample components to facilitate research into different smaller sub-groups: a General Population Sample, an Ethnic Minority Boost Sample, an Immigrant and Ethnic Minority Boost Sample, and the British Household Panel Survey Sample^2^. The main adult questionnaire^3^ for waves 4 and 10 was an interviewer-administered CAI questionnaire (either face-to-face using the CASI module or by telephone). The questionnaire was administered to all eligible household members aged sixteen or above who responded to a wide range of questions covering: family and households, income, education, politics, education, health and wellbeing, and attitudes.

The UKHLS in waves 1, 4, and 10 contained questions regarding attitudes and beliefs toward the seriousness, urgency, and preventability of climate change. We used data from waves 4 and 10 because they were more recent and provided consistent and more fine-grained measurements (5-point Likert scales compared to dichotomous responses in wave 1). Wave 4 (covering 2012 to 2014) and wave 10 (covering 2018 to 2020) contained responses from 38037 individuals (valid rate = 80.81%) and 31498 individuals (valid rate = 91.78%) respectively, after listwise deletion of cases with missing values in the attitudes to risks of climate change variables. Missing data analysis—including univariate missingness and multivariate missing data pattern analysis—showed that missingness of climate change risk attitudes variables in both waves 4 and 10 was plausibly *Missing At Random* (MAR; for more details of missing data diagnosis, see Supplementary Appendix A), which allowed the estimation of models to use maximum likelihood.

### Measures of attitudes to climate change risk

We selected five statements^4^ regarding opinions on climate change, similar to those used by Rhead et al. (2018). Participants indicated their agreement with each statement using either a 5-point Likert scale (1 = Strongly disagree to 5 = Strongly agree) or a dichotomous response (0 = No and 1 = Yes). These items captured attitudes toward the risk of climate change and its impacts on the UK in the future (see Table 1).

**Table 1:** Measures of ACCRs.

Variable name	Statement
Beyond control	Climate change is beyond control, it’s too late to do anything about it.
Too far in future	The effects of climate change are too far in the future to really worry me.
Affected within 30 years	People in the UK will be affected by climate change in the next 30 years.
Major disaster	If things continue on their current course, we will soon experience a major environmental disaster.
Crisis exaggerated	The so-called ‘environmental crisis’ facing humanity has been greatly exaggerated.

### Statistical analyses

We carried out *k*-means cluster analysis to partition people’s attitudes into homogeneous clusters. We used the three criteria proposed by Makles (2012) to decide on the optimal number of clusters: (i) The number of clusters associated with the first non-linear inflection point when plotting the within-cluster sums-of-squares (WSS) in order of size, and (ii) The number of clusters with the largest ratio of WSS to total sums-of-squares (TSS), referred to as the *η*^2^ coefficient and (iii) the proportional reduction of error (PRE) coefficient (Schwarz, 2008), which measures the proportional reduction of WSS for a solution with *k*-clusters compared with one with K-1 clusters. Further cluster comparison and validation was conducted by a one-way-analysis-of-variance (ANOVA) of the differences of items’ means across clusters.

We assigned each participant to membership of the cluster with the nearest cluster centroid in the optimal solution, and then used multinomial logistic regression analysis to predict cluster membership based upon characteristics of the respondent. The characteristics we used were age, sex, education, income, and political affiliation. Age and income were mean-centred.

## Results

### Descriptive statistics

Descriptive statistics of ACCRs and social characteristics (age, income, sex, education, and political affiliation) are shown in Table 2. Sex was treated as dummy variable (1 = male, 0 = female). Age was coded as a continuous variable, ranging from 16 to 100 in wave 4 and 16 to 103 in wave 10. Income was captured by people’s gross monthly personal income in thousands of UK pounds, ranging from −8.85 to 27.47 in wave 4 and −3.33 to 26.63 in wave 10^5^. Education was an ordinal variable with five categories, ranging from 1 = no qualification to 5 = degree^6^. Political orientation^7^ was coded according to the party the respondent feels closest to. In wave 4 data, we coded political affiliation as, 0 = Right-wing (Conservative, Ulster unionist, Democratic unionist), which is used as the reference category, 1 = Left-wing (Labour, Liberal democrat, Scottish National Party, Plaid Cymru, Green Party, Social Democratic and Labour Party, Alliance Party, Sinn Fein), 2 = Other Parties (None of the above). In wave 10 data, several parties have been removed in the questionnaire (i.e., Democratic unionist and Social Democratic and Labour Party) while some have been added (i.e., UK Independence Party, The Brexit Party, and Change the UK). Accordingly, we coded political affiliation as, 0 = Right-wing (Conservatives, Ulster unionist, UK Independence Party, The Brexit Party), which is used as the reference category, 1 = Left-wing (Labour, Liberal democrat, Scottish National Party, Plaid Cymru, Green Party, Alliance Party, Sinn Fein), 2 = Other Parties (None of the above).

**Table 2:** Descriptive statistics of ACCRs and demographic variables in waves 4 and 10.

ACCRs	Mean		SD		*N*		Min		Max	
	W4	W10	W4	W10	W4	W10	W4	W10	W4	W10
Major disaster	3.31	3.65	1.00	0.96	38,037	31,498	1	1	5	5
Crisis exaggerated	3.02	2.58	1.03	1.05	38,037	31,498	1	1	5	5
Beyond control	2.65	2.48	0.98	0.95	38,037	31,498	1	1	5	5
Too far in future	2.64	2.36	1.05	1.04	38,037	31,498	1	1	5	5
Affected within 30 years	0.78	0.84	0.41	0.36	38,037	31,498	0	0	1	1

Demographic variables	Mean		SD		*N*		Min		Max	
	W4	W10	W4	W10	W4	W10	W4	W10	W4	W10
Age	47.13	49.65	18.04	18.57	37,878	30,981	16	16	100	103
Income (£1000)	1.67	1.91	1.53	1.70	37,878	30,981	−8.85	−3.33	27.47	26.63

Demographic variables	Categories				*N*		Percent			
					W4	W10	W4		W10	
Sex	Female				21,076	17,206	55.64		55.54	
	Male				16,802	13,775	44.36		44.46	
Education	No qualification				4405	2720	11.63		8.77	
	GCSE etc				11,608	8711	30.65		28.12	
	A-level etc				8252	6625	21.79		21.38	
	Other higher education				4516	3889	11.92		12.55	
	Degree				9097	9036	24.02		29.17	
Political affiliation	Right-wing				6729	1493	36.21		38.43	
	Left-wing				11,145	2374	59.98		61.11	
	Other party				707	18	3.80		0.46	

### *k*-means cluster analysis

**Fig. 1:**
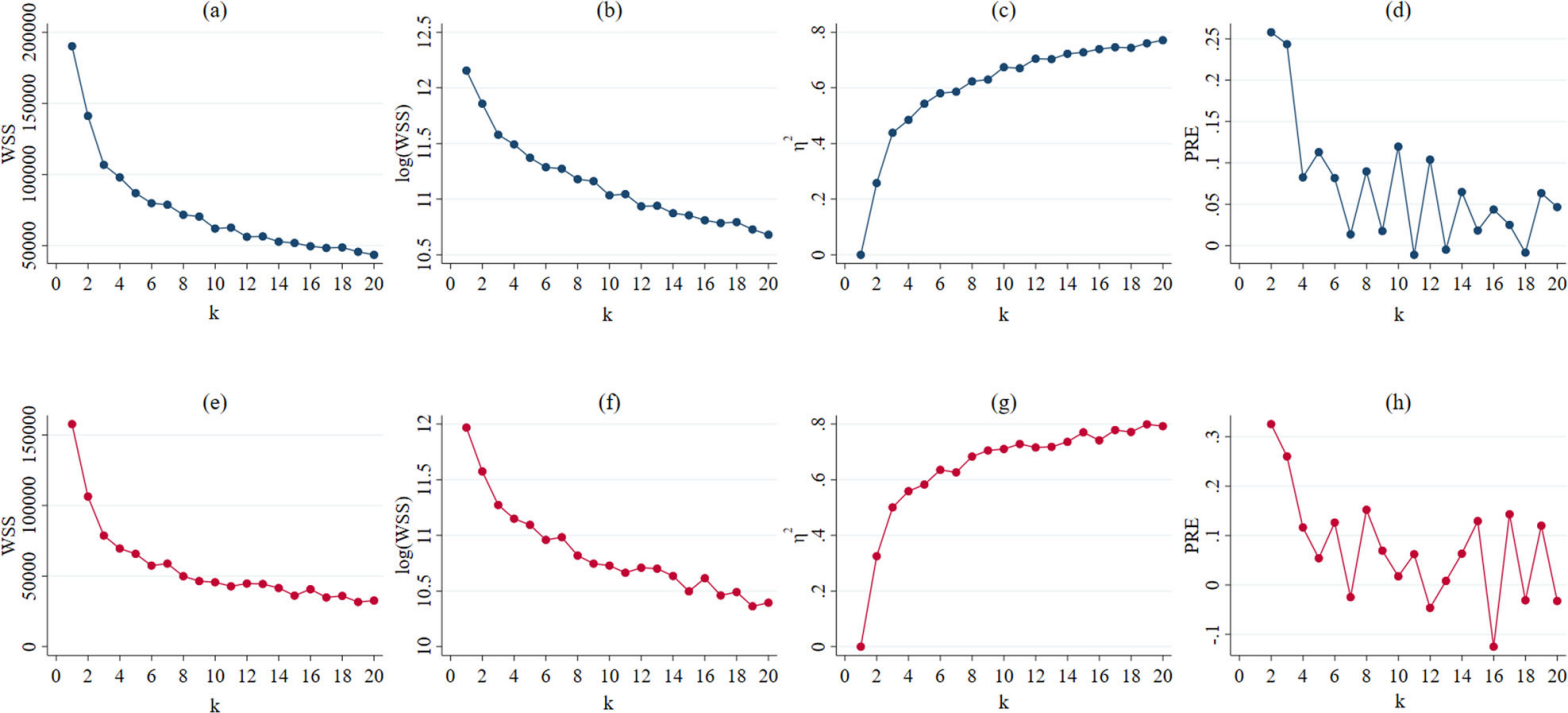
WSS, log (WSS), *η*^2^, and PRE for all *k*-cluster solutions in waves 4 and 10. **a**–**d** displayed wave 4 data. **a** WSS (within sum of squares), **b** *log* (WSS), **c** *η*^2^, and **d** PRE (proportional reduction of error) for 20 cluster solutions. **e**–**h** displayed wave 10 data. **e** *WSS* (within sum of squares), **f** *log* (*WSS*), **g** *η*^2^, and **h** PRE (proportional reduction of error) for 20 cluster solutions. *k* stands for the number of cluster.

**Fig. 2:**
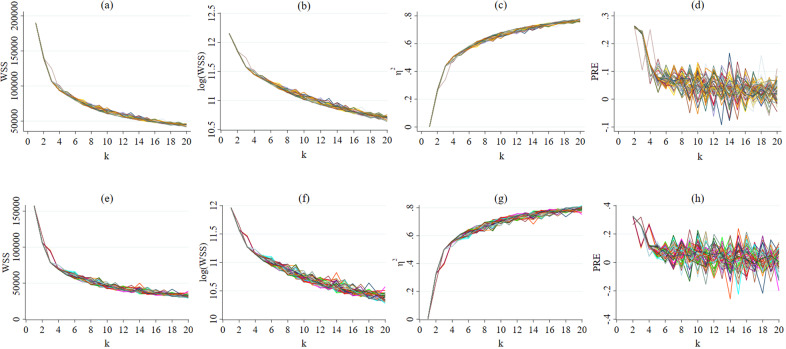
Fifty different WSS, log (WSS), *η*^2^, and PRE curves for *k* = 20. **a**–**d** displayed wave 4 data. **a** *WSS* (within sum of squares), **b** log (WSS), **c** *η*^2^, and **d** PRE (proportional reduction of error) for 20 cluster solutions (*k* = 20). **e**–**h** displayed wave 10 data. **e** WSS (within sum of squares), **f** log (WSS), **g** *η*^2^, and **h** PRE (proportional reduction of error) for 20 cluster solutions (*k* = 20). Lines in 50 different colours indicate different cluster solutions by repeating the clustering 50 times with different starting points.

*k*-means results showed that clustering with *k* = 3 is the optimal solution in both waves 4 and 10. At *k* = 3, a kink or cut-off point occurred in both the WSS and log (WSS) figures (see Fig. 1), where the difference in the within-cluster dissimilarity is not substantial. Compared to the *k* = 2 solution, $\eta _3^2$ pointed to a 44% reduction in the WSS and PRE_3_ pointed to a reduction of about 24% in wave 4 data, while $\eta _3^2$ pointed to a 50% reduction in the WSS, and PRE_3_ pointed to a reduction of about 26% in wave 10 data. The reduction in WSS was negligible for *k* > 3. Sensitivity analyses showed that *k* = 3 was robust. Repeating the analysis 50 times with randomly selected cluster centroid starting points resulted in *k* = 3 as the optimal solution 49 times, with *k* = 4 once in wave 4 data; *k* = 3 as the optimal solution 47 times, with *k* = 2 twice and *k* = 4 once in wave 10 data (see Fig. 2). Results of the ANOVAs with two waves data both showed that there were significant differences between the three clusters to all attitude questions (*Major Disaster*_wave4_ (*F*(2, 38034) = 5056.80, *p* < 0.001), *Crisis Exaggerated*_wave4_ (*F*(2, 38034) = 8254.75, *p* < 0.001), *Beyond Control*_wave4_ (*F*(2, 38034) = 7379.56, *p* < 0.001), *Too far in Future*_wave4_ (*F*(2, 38034) = 14656.25, *p* < 0.001), *Affected within 30 years*_wave4_ (*F*(2, 38034) = 553022.80, *p* < 0.001; *Major Disaster*_wave10_ (*F*(2, 31497) = 8782.13, *p* < 0.001), *Crisis Exaggerated*_wave10_ (*F*(2, 31497) = 13973.79, *p* < 0.001), *Beyond Control*_wavw10_ (*F*(2, 31497) = 5151.37, *p* < 0.001), *Too far in Future*_wave10_ (*F*(2, 31497) = 12776.86, *p* < 0.001), *Affected within 30 years*_wave10_ (*F*(2, 31497) = 970.00, *p* < 0.001).

**Fig. 3:**
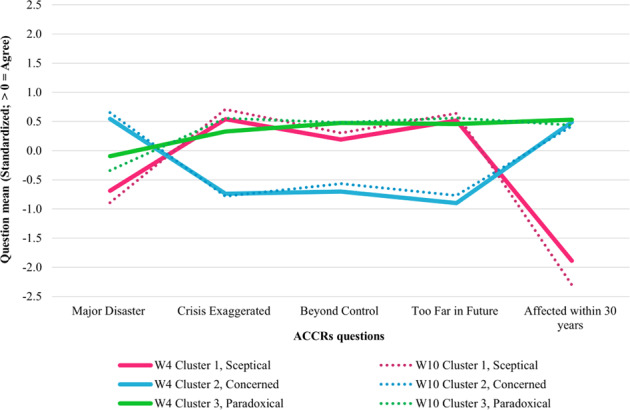
Item means for the three-cluster model (Sceptical_W4_ = 21.3%, Concerned_W4_ = 35.4%, Paradoxical_W4_ = 43.2%; Sceptical_W10_ = 15.6%, Concerned_W10_ = 43.8%, Paradoxical_W10 _= 40.7%). W4, wave 4. W10, wave 10.

A key finding is that the three-cluster pattern is almost identical in the two waves (see Fig. 3). Members of cluster 1 disagreed most strongly that people will be affected by climate change in the next thirty years compared to the other two clusters (see Fig. 3). They had the strongest propensity to embrace a negative statement of climate change risk. For example, when considered against people’s outlook towards the trend of climate change, members would be less likely to worry about the effect of climate change in the future and more likely to negate the action to cope with climate change. In this sense, members of cluster 1 are most optimistic about the risk of climate change and therefore make light of potential major environmental disasters on various grounds, such as that climate change is exaggerated and distant. Therefore, it is likely that cluster 1 is the group that has sceptical thinking. This corresponds to the sceptical cluster identified by other studies (see e.g., Dunlap, 2013; Maibach et al., 2011; Sibley and Kurz, 2013). In effect, the defining feature of sceptical thinking is to downplay the risk. Hence, we named cluster 1 “Sceptical”.

Members of cluster 2 have an overall lowest probability of agreeing with the three negative statements and the highest probability of agreeing with the two positive statements. People in this cluster tend to agree that problems are coming for both the environment (Major disaster) and climate (Affected within thirty years) and tend to agree that the environmental crisis truly exists and has not been exaggerated. Moreover, members of cluster 2 indicate concern about climate change risk. For instance, this cluster concurred that climate change is not too far in the future to worry about nor beyond control, implying their potential approval of urgent efforts to tackle with climate change. Given this, cluster 2 is the group that are concerned about climate change risk and the need to deal with it urgently. We named cluster 2 “Concerned”.

Members of cluster 3 seem undecided as to what position they stand for. This cluster finds the effects of climate change are too far in the future to worry them but also likely to come within thirty years. From the point of attitudes pattern, they neither agreed with the positive nor disagreed with negative statements, ranging from −0.10 to 0.53 in wave 4 and −0.34 to 0.56 in wave 10. Item probabilities for cluster 3 suggest that members, on the one hand, possess a similar attitude pattern with cluster “Sceptical” regarding the negative cognitive evaluation of climate change, such as crisis facing humanity has been exaggerated. On the other hand, cluster 3 is far less likely to disagree that the problems of climate change are coming compared to cluster “Sceptical”. For instance, they displayed the highest probability to agree that people will be affected by climate change in the near future. However, this cluster showed the strongest inclination to agree that climate change is out of control and it is too late to do anything about it, implying they do feel powerless and passive as the risk is going to happen to an extent but they do not believe there is much can be done. Note that members of cluster 3 also modestly disagree that major environmental disasters will occur though it is weak in strength, indicating they feel climate change is probably overblown. To sum up, this cluster appears to be expressing ambivalent risk attitudes. Given this, we labelled it “Paradoxical”. This group is consistent with Rhead et al. (2018) environmental concern cluster with the same name and similar pattern.

### Multinomial logistic regression analysis

We fitted two multinomial logistic regression models in each wave, one treating education as continuous and one as dummy-coded categories. We chose the latter model as it had a lower Akaike’s Information Criterion (AIC; 77395.93 with 20 parameters for categorical and 77,437.46 with 14 parameters in wave 4, 60,062.28 with 20 parameters for categorical and 60120.85 with 14 parameters for continuous in wave 10). Table 3 shows the parameter estimates for the multinomial logit model^8^.

**Table 3:** Odds ratios for variations in sex, age, education, income, and political affiliation by ACCRs clusters in waves 4 and 10.

	Sceptical				Paradoxical				Concerned			
	Odds-ratio		*p*		Odds-ratio		*p*		Odds-ratio		*p*	
	W4	W10	W4	W10	W4	W10	W4	W10	W4	W10	W4	W10
Male	1.428	1.524	0.000	0.000	1.110	1.168	0.000	0.000	–	–	–	–
Age	1.001	0.995	0.437	0.000	1.005	1.000	0.000	0.888	–	–	–	–
Education												
No qualification	–		–		–		–		–	–	–	–
GCSE etc	0.553	0.526	0.000	0.000	0.598	0.595	0.000	0.000	–	–	–	–
A-level etc	0.382	0.351	0.000	0.000	0.453	0.436	0.000	0.000	–	–	–	–
Other higher education	0.356	0.270	0.000	0.000	0.409	0.410	0.000	0.000	–	–	–	–
Degree	0.193	0.143	0.000	0.000	0.251	0.205	0.000	0.000	–	–	–	–
Income	0.935	0.920	0.000	0.000	0.967	0.953	0.000	0.000	–	–	–	–
Political affiliation												
Right-wing	–	–	–	–	–	–	–	–	–	–	–	–
Left-wing	0.428	0.269	0.000	0.000	0.633	0.413	0.000	0.000	–	–	–	–
Other party	1.048	0.377	0.657	0.225	0.943	0.562	0.546	0.270	–	–	–	–
Constant	1.811	1.022	0.000	0.830	2.907	2.482	0.000	0.000	–	–	–	–

Note: W4, wave 4. W10, wave 10.

The two waves of data manifested consistent predicted probabilities of ACCRs cluster membership by socio-demographic characteristics except for age. In wave 4 data, age differences were not a good predictor of membership of the Sceptical versus the Concerned cluster, but higher age was associated with belonging to the Paradoxical versus the Concerned cluster: about 5% more likely per decade of age difference. Conversely, wave 10 data suggested that lower age was associated with belonging to the Sceptical versus the Concerned cluster—about 5% more likely per decade of age difference—but age was no longer a significant predictor of membership of the Paradoxical versus the Concerned cluster.

Males were around 50% percent more likely than females to belong to the Sceptical cluster and over 10% more likely to belong to the Paradoxical cluster (11% in wave 4 and 16.8% in wave 10) compared to the Concerned cluster. Higher income was associated with belonging to the Concerned cluster versus either of the others, by 6.5% (wave 4) and 8% (wave 10) more likely per £1000 of income compared to the Sceptical cluster and 3.3% (wave 4) and 4.7% (wave 10) more likely compared to the Paradoxical cluster.

The strongest predictors of cluster membership were education and political affiliation. The higher the level of qualifications, the more likely participants were to belong to the Concerned cluster and less likely they were to belong to the other clusters in two waves. The biggest difference between adjacent education levels was between those with no qualifications versus those with age-16, school-level qualifications (GCSE etc.). The latter were at least 40% (in wave 4; 40.5% in wave 10) more likely than those with no-qualifications to belong to the Concerned cluster compared to either of the others. Across the full range of qualifications, those with a degree were at least 70% (in wave 4, 79.5% in wave 10) more likely than those with no qualifications to belong to the Concerned cluster compared to the other clusters. Regarding political affiliation, those aligned with left-wing parties were at least 36.7% in wave 4 and 58.7% in wave 10 more likely than right-wingers to belong to the Concerned cluster compared to the other clusters. Supporters of other parties were largely aligned with right-wingers in wave 4 and left-wingers in wave 10 with regard to cluster membership.

**Fig. 4:**
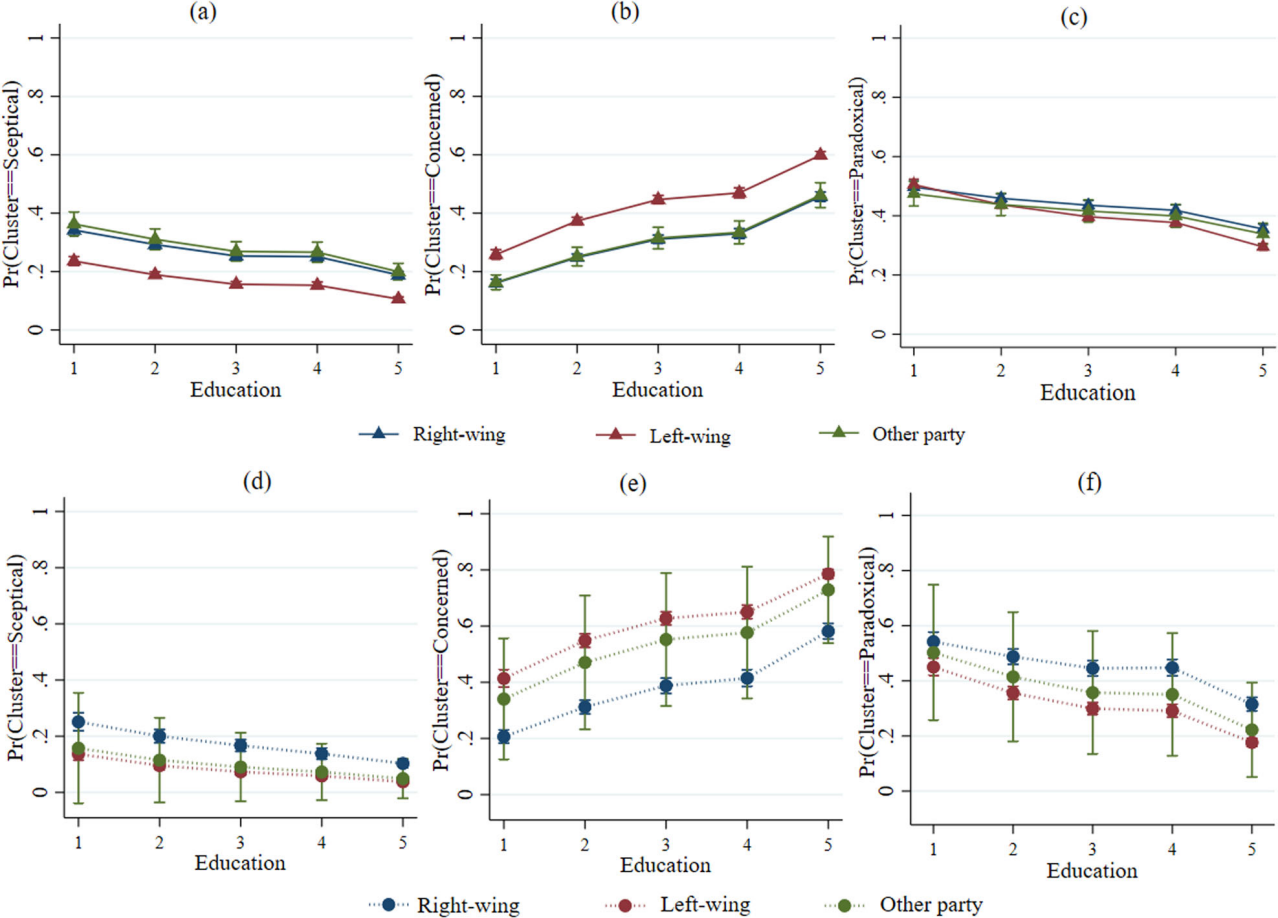
Predicted marginal probabilities of political affiliation with 95% CI at educational level on three ACCRs clusters in waves 4 and 10. **a**–**c** displayed wave 4 data. **a** Sceptical, **b** Concerned, and **c** Paradoxical. **d**–**f** displayed wave 10 data. **d** Sceptical, **e** Concerned, and **f** Paradoxical. 1 = No qualification, 2 = GSCE etc., 3 = A-Level, 4 = Other higher education, 5 = Degree.

Figure 4 shows the marginal probabilities of cluster membership for the different levels of education and political affiliation. There was a clear distinction between supporters of left-wing parties vs. right-wing parties with regard to membership of the Sceptical (panel a) and the Concerned (panel b) cluster in wave 4 data, as well as membership of three clusters (panels d, e, f) in wave 10 data. In each case, cluster membership was strongly associated with education, negatively for the Sceptical and Paradoxical cluster. In each wave, education was substantially associated positively with membership of the Concerned cluster, the differences associated with political affiliation were much bigger for this cluster than the others^9^.

### Transitions in ACCR clusters between wave 4 and wave 10

Although cluster structure was very similar in the two waves, the overall proportions of each cluster have changed. The Paradoxical (43.2%, Concerned = 35.4%, Sceptical = 21.3%) was the biggest cluster in wave 4, while the Concerned (43.8%; Paradoxical = 40.7%, Sceptical = 15.6%) has become the biggest in wave 10. Table 4 gives the transition matrix of group members who shift attitudes to climate change risk from wave 4 to wave 10. Results indicated that 71.6% of the population in group Concerned in wave 4 continue to be members of the Concerned cluster risks in wave 10. By contrast, over half of the population of the Paradoxical in wave 4 shifted attitudes to the Concerned in wave 10, around one-fifth people in group Sceptical moved to the Concerned in wave 10. It is also noteworthy that nearly half of the Sceptical cluster shifted to the Paradoxical cluster (43.2%) in wave 10, and over one-fifth of the Concerned cluster also moved to the Paradoxical cluster (23.8%).

**Table 4:** Transition matrices by three clusters across waves 4 and 10.

	Wave 10 (2018–2020)						
		Cluster	Sceptical	Concerned	Paradoxical	Missing	Total
Wave 4 (2012–2014)		Sceptical	1595 (37.1%)	843 (19.6%)	1858 (43.2%)	300	4596
		Concerned	394 (4.7%)	6036 (71.6%)	2003 (23.8%)	260	8693
		Paradoxical	1190 (13.2%)	3008 (33.3%)	4848 (53.6%)	590	9636
		Missing	518	1002	1230	628	3378
		Total	3697	10,889	9939	1778	26,303

To summarise, we observe a considerable movement between the clusters with a general trend towards higher membership of the Concerned cluster, but the overall cluster structure appears to be robust over time.

## Discussion

### General discussion

This study segmented respondents based on their appraisal of statements of climate change risk. Three discrete clusters were suggested by the application of *k*-means cluster analysis using the within-sum-of-squares statistic: Sceptical, Concerned, and Paradoxical. The proportions, item probability patterns of the three segments, and transition by clusters over time express Britain’s dynamic pattern of ACCRs. Our multinomial logistic regression analysis of the socio-demographic profile of cluster members has found a variation by sex, age, education, income, and political affiliation between the three clusters.

Members of the Sceptical cluster deny that climate change is going to be problematic. This is consistent with the “Sceptical”, “Doubtful” and “Denial” groups identified in previous research (e.g., Dunlap, 2013; Kácha et al., 2022; Maibach et al., 2011; Sibley and Kurz, 2013; Whitmarsh, 2011), this group minimise the projected effects of climate change and therefore is less likely to support actions to mitigate those effects. Different from outright scepticism (thinking that denies the existence of climate change), their doubts are focused on the seriousness, necessity, effectivity, and urgency of tackling climate change. This aligns with previous observations that absolute denial about the existence of climate change is relatively rare in the UK (e.g., Poortinga et al., 2011; Taylor et al., 2014; Whitmarsh, 2011). On the one hand, the Sceptical shares some similarity with apathetic attitudes in the *Disengaged* class found in Rhead et al. (2018) study; both do hold optimistic views about—and dismissive attitudes toward—climate threats. For the Sceptical cluster, climate change seems distant, happening mostly in the future and to other people. This is potentially indicative of optimistic temporal and spatial biases in their perception of climate change threats and therefore appear to dampen enthusiasm for tackling climate change (see Gifford et al., 2009 for a discussion of optimistic temporal and spatial biases). Given that pessimistic affective messages in climate change appeals could increase risk perception and facilitate engagement with climate issues more than optimistic affective messages (Morris et al., 2020), future studies examining the effect of possible optimism bias on sceptical thinking about climate change risk may prove valuable.

The Concerned cluster is the most convinced that climate change is happening and has not been exaggerated, which is strongly indicative of potential approval of urgent climate change mitigation efforts and being ready to engage with climate change (e.g., Maibach et al., 2011; Roser-Renouf et al., 2014). A large proportion of the Concerned cluster (35.44% of all respondents) do not agree that climate change is too far in the future and not worth being concerned about, making them similar to the “Alarmed” and “Concerned” groups identified in previous studies (see also Maibach et al., 2011; Metag et al., 2017; Myers et al., 2012; Poudyal et al., 2020). A similar climate attitude group has also been found in the US (Goldberg et al., 2020). We note that Leiserowitz et al. (2009) found that people who are concerned about the issue use media channels to capture the information about climate change. This view was supported by Wonneberger et al. (2019), who found that those concerned about climate change paid more attention to media coverage of the COP21 meeting than did those who were doubtful about climate change. However, Rosenthal (2022) found that individuals’ worldviews reflected their personal experience of climate change, which can be influenced by information sources such as mainstream and social media. These associations could be explored as a way to persuade the Sceptical segment.

The Paradoxical cluster displayed a mixed ACCR profile; they had the highest probability of agreeing that people will be affected by climate change in the next thirty years and also agreed with the statement that it was too far in the future to worry about. Consistent with Rhead et al. (2018) study, the Paradoxical class recognised environmental problems but then seem to dismiss or trivialise them. The Paradoxical cluster appear confused and perhaps less capable of dealing with threats that are happening in the future with drawn out and uncertain impacts with complex causality (Marshall, 2005). The Paradoxical group in our study was the largest (43.22% of the respondents) in wave 4 and just second largest by a narrow margin in wave 10, showing that many in Britain still feel ambivalent and/or confused about climate change risk. This corresponds to Kácha et al.’s (2022) findings (in their study of 23 Europe countries)—where is the Indifferent group—which is the segment similar to the Paradoxical cluster who hold “neither-nor” attitudes to negative and positive statements of climate change. Rhead et al. (2018) explored the association between attitudes and pro-environmental behaviours and found that the odds of “Paradoxical” class members engaging in pro-environmental behaviours were lower than the “Pro-environment” and the “Neutral Majority” classes.

As well as differing in ACCRs, the clusters also differ in socio-demographics. Compared to females, males had a significantly higher probability of being the Sceptical and Paradoxical clusters, and a lower probability of being the Concerned cluster. There are many potential explanations for this tendency, for example, females are found to have more nurturing and maternal natures and more altruistic values; they also tend to have a longer time horizon (see e.g., Coelho et al., 2017), thinking about how the environment will be for the next generation rather than merely how it is at present (e.g., Milfont et al., 2015; Smith et al., 2017). Another explanation focuses on the origins of racial and gender variance in risk perception, as being rooted in cultural identity-protective cognition. To generalise, the theory claims that women are more worried about risks, including the risks associated with environmental pollution and the climate crisis, than men because the statements of risk challenges the cultural identities of hierarchical and individualistic males—especially white males (Kahan et al., 2007). This is consonant with the findings that conservative white males contribute significantly to climate change denial (McCright and Dunlap, 2011a).

Regarding age, younger respondents were more likely to belong to the Concerned versus the Paradoxical cluster in wave 4. Wave 10 data suggested that younger respondents were more likely to be in the Concerned versus the Sceptical cluster. This implies that younger people hold more concerned attitudes to climate change risks than the older ones. In line with Marks et al. (2021), our speculation is that young people may be more concerned because of worries about what harms they will be left to cope with while currently having little power to limit those harms. Older participants were more likely to belong to the Paradoxical cluster in wave 4 and the Sceptical cluster in wave 10. For the older participants, perhaps future climate change is a smaller worry for them relative to—for example—current personal health issues. Another possible element of this complex picture is that worrying about climate change and being willing to tackle it is a relative threat to older people who stand to lose the most because their social status is most entrenched in traditional worldviews (Goto Gray et al., 2019; Liere and Dunlap 1980). We also found that the higher one’s income, the lower probability of being the Sceptical and Paradoxical cluster over the Concerned cluster. One well-known explanation rests on the assumption that concern about environmental quality has some of the properties of a luxury good, which can be indulged only after more basic material needs such as adequate food, shelter, and economic security are met (see e.g., Liere and Dunlap, 1980; Maslow, 1970).

Traditional societal fault lines of sex, age and income seem to have some relationships with ACCRs, but these orthodox predictors were overshadowed in predictive power by education and political affiliation; the odds ratios of education and political affiliation were consistently the highest across the two waves data compared to other social characteristics. Regarding education, we saw that higher educational attainment increases the likelihood of belonging to the Concerned cluster versus the other two clusters compared to no qualification. We might theorise that the more educated a person is, the more able that person is to process complex information and rationally assimilate scientific evidence about climate change (Hoffmann and Muttarak, 2017). Another speculation is that people who have higher education may be more directly exposed to learning about climate change, which develops their climate change awareness. It seems reasonable to assume that exposure to climate science typically increases with education (Bohr, 2014), and higher education also brings raised exposure to political ingroup norms (Ehret et al., 2017); Although we note that Kahan (2017) has observed that science comprehension may be not necessarily correlated with beliefs about climate change. Hence, right-wingers are more inclined to be motivated to defend ideological positions associated with their conservative worldviews when confronted with information about climate change and climate-related crises. Similarly, individuals with left-wing political affiliations are more likely to be concerned; this confirms the finding of Milfont et al. (2015). Other researchers have also found that an interaction of political affiliation with education to be a good predictor of citizen concern about climate (e.g., Hamilton, 2011; McCright, 2011). However, in this study, we found no such interaction and that the higher level of education that a person has, the less likely they are to belong to the Sceptical and Paradoxical clusters regardless of their political orientation. Similarly, the effect of political orientation is independent of education.

While these co-variates do seem to be critical in distinguishing people’s ACCRs, sex, age, income, education, and political affiliation are not meant to provide an explanation nor are the list of included predictors meant to be exhaustive. Other potential socio-demographic predictors should also be considered in the future, including social class or grade. At the same time, the potential structural effect of age on education, income, and other variables directly related to the age variation is worth noting^10^. For example, the youngest respondents in our sample were aged 16, but the minimum age for the top category of education is 21. Therefore, there might be a small structural age effect of education. Attitudes are not fixed and might change throughout a person’s lifetime, perhaps driven by their country’s climate policy or mitigation targets, and indeed we observed a lot of movement in the respondents’ attitudes across two waves. Given that this is a fixed set of respondents who are aging together we cannot completely distinguish between age and period effects with these data. However, given that within the data older people tend to be more sceptical, we speculate that the nett shift evidenced here is probably a period effect—one affecting the whole population. However, this overall trend belies a great deal of turbulence with individuals shifting between all three clusters. Therefore, it would be meaningful to examine the characteristics of people who changed in one direction rather than the other in future work. Beyond exploring socio-demographic predictors, the question of how values, worldviews, ideology, and identity (shaped by income, social class, education, and political affiliation) further influence climate change attitudes warrants an in-depth investigation. Previous literature has indicated that climate scepticism is rooted in people’s core values and worldviews, mirroring the findings that identity-protective, cognition-driven political affiliation and education are the strongest correlates of denial and/or dismissive attitudes to climate change (Poortinga et al., 2011). Additionally, future research could usefully examine other explanatory concepts including the effects of historical events and individual experiences of extreme weather, which might also predict people who moves between clusters.

### Implications

In this paper, we make a conceptual contribution through introducing a typology of ACCRs using *k*-means cluster analysis on a national probability sample and explore the cross-time transitions of cluster membership, thereby diversifying the evidence base beyond cross-sectional factor and latent class analysis. We extend existing literature on population classification by tracing the UK public ACCRs cluster trajectories between 2012 and 2020. Leiserowitz et al. (2011) assumed constant segmentation structure, based upon six Americas segmentation measured at time one, and explored how segmentation size changed over time without evaluating if the segmentation structure was applicable to the actual data at each time point. In contrast, we have gone a step further by evaluating the applicability to real-world data by data-driven *k*-means clustering, where the cluster solution is constructed independently at each wave. Cluster patterns in our study were consistent in 2012 and 2020, even though at the individual level there was significant movement between clusters. This combination—stable patterns but mobile individuals—suggests a lot more fluidity of opinion than perhaps media debates might suggest and this opens up the possibility of targeting policy interventions and creating information campaigns that are tailored towards the needs of specific groups with different risk attitude patterns.

Our findings suggest that targeting the Paradoxical populations should be prioritised when developing communication strategies. The Paradoxical, the largest cluster during the period of wave 4 (2012 to 2014) still included over 40% of population at wave 10 (2018 to 2020). However, membership of the Paradoxical cluster is least stable and the significant movement from the Paradoxical to the Concerned cluster between waves 4 and 10 suggest that messages targeted at those with paradoxical beliefs may be most effective at causing significant shifts in overall ACCRs. It is noteworthy then that some degree of ambivalence, mixed feelings, or non-straightforward sceptical thinking appear to be widespread in the UK, which might be indicative of people needing more or perhaps differently framed information to form clear opinions. Since the Paradoxical tend to be worried about climate change but feel powerless to cope with, it could be that the climate change affective messages for the Paradoxical should be motivation-oriented with pessimistic ending because it could trigger higher engagement with the issue than optimistic ending (see Morris et al. (2020) for a recent study of the issue of the relationship between affect, efficacy and climate change risk perception).

The Sceptical often questioned the uncertainty of climate change and may need to see risk quantifications to move them to middle ground. Since climate information or consultants directly or indirectly influence how people judge and assess risk, climate scientists may consider quantifying the risk and visualising the mitigation efforts in order to communicate with both ends of that spectrum the very real damage that climate change may inflict (Wendel, 2016) and translate scientific and local knowledge in climate change risk reduction to realities on the ground (Trogrlić et al., 2021). Scientific messages for the Sceptical cluster may also consider providing news of climate disasters and informative mitigation action policy as previous research has found that dire risks and climate apocalypse can enhance public engagement with climate change issues (see e.g., Mayer and Smith, 2019). Yet, it would be tiresome to hear every effect of climate change put on the worst-case scenario of death and destruction; messages with strong emotional content may erode intention to act among those who were not already highly concerned about climate change (Kerr, 2007; McNeish, 2017). Tailored risk communication messages may focus not only on how to best represent key information and messages to evince behaviour change in general but also on how to tailor such information to the segments and then disseminate it through appropriate channels in order to reach each segment (Andor and Fels, 2018; Rosenthal and Linder, 2021). There is now a significant body of research in communication studies that suggests that information channels, platforms, news outlets and media types can vary widely by demographic profiles and population segments (see, for example, Althaus et al., 2009; Diehl et al., 2019; Dutta-Bergman, 2004; Shehata and Strömbäck, 2021; Vara-Miguel, 2020). Hence, the relationship between population classes of ACCRs attitudes, patterns of media use, trust in different sources, and information seeking behaviours warrant exploration in future research. In sum, tailoring climate change information and communication for different clusters within the UK public may help narrow the divide on attitudes to climate change risks and foster greater consensus towards pro-environmental views.

### Limitations

Despite these important insights, our study has several limitations.

The use of secondary data does create ontological constraints. The data were designed to be used in the context of a broad national survey in which climate change questionnaire is only one module of the whole interview. Consequently, the survey is not a perfect fit to our requirements. Some nuance is lost about details, which are not included in the survey for example regarding a respondent’s willingness to cope with climate change risk.

A second issue is that a relatively small number of directly matching survey items also drove the modest compromise of including two items that did not explicitly mention climate change: the statements about the “environmental disaster” and “environmental crisis”. It is possible that respondents may view environmental hazards as referring to other factors (e.g., plastic pollution, or roadside particulates) rather than specifically climate change issues. However, we would argue (i) that the appendage of the terms “disaster” and “crisis” will inevitably bring to mind the most talked about environmental challenge and (ii) in the survey itself, climate change is the individual issue that warranted the most individual questions. It is reasonable to posit that the impacts of climate change are the most salient aspects of “environmental disaster” and the “environmental crisis” for the respondents when they are responding to these questions. However, we acknowledge that this is an assumption, which we have no way of explicitly testing. These types of compromises are of course not unique to this study; all secondary analysis faces this issue. Future studies constructed with the specific purpose of audience segmentation of risk perception of climate change would be a natural remedy for this.

A third limitation is the exclusive reliance on risk attitudes variables solely, rather than a set of measurement such as knowledge, policy preferences, and behavioural responses. Although this avoided confounding attitudes towards risks of climate change with other climate literacy issues and producing results relating to the behavioural dimension by introducing additional variance into the analysis, this also oversimplifies attitudes that are multi-faceted and complex. For example, an intervention based around increasing people’s knowledge of the scientific evidence is only relevant if they do not have that knowledge. Undoubtedly, such variables do have a relationship with climate change attitude cluster membership and introducing them into the analysis may increase the predictive power of behaviour change models (Metag and Schäfer, 2018). Future research may consider using a broader array of constructs to draw a whole picture of climate change risk attitudes typology. Although cluster analysis is an effective method for identifying homogenous groups (with the underlying assumption that distinct groups exist), the approach is not entirely objective. When performing the statistical procedures, some subjective decisions were nevertheless required. For example, the selection of which variables to use as input, the algorithms, and the centroids for the k-clusters (see Hartigan and Wong, 1979).

It is also worth mentioning that method biases arise from having a common rater, a common measurement context, and the characteristics of the climate change risk attitudes items themselves are likely to be a powerful in influence people’s responses. For example, face-to-face interviews—as employed by the UKHLS—may induce consistency motif issues, and lower accuracy than self-completed questionnaires. Interviewer characteristics, expectations, and verbal idiosyncrasies may also cause response biases (Podsakoff et al., 2003). Lastly, the results are specific to the UK population and may not generalise to other countries. Specifically, environmental concern within developing countries may have very different structures. Future comparative studies may pay attention to variation in the typologies of ACCRs between developed and developing countries.

## Conclusion

This study produced a classification of ACCRs as they were during a period when the UK slashed carbon emissions. We used survey data from the UKHLS to place people into three discrete segments using *k*-means cluster analysis: Sceptical, Concerned, and Paradoxical. The Sceptical group, see climate change threats as overblown and agree with the statement that the crisis has been exaggerated though still recognising the existence of climate change. The Concerned group displayed an overwhelmingly urgent worry about climate change risks. The Paradoxical group did not indicate any active attitudinal response toward planning for climate threats though they admit that major environmental disasters might occur if things continue as they are. The overall cluster structure was maintained over the period through 2012 to 2020, however, the members of Paradoxical and Sceptical tended to move toward the Concerned cluster between the two waves of data that we analysed. It is also noteworthy that despite the fact of transition toward concerned attitudes, the Paradoxical group still makes up nearly half of the British population making it worth exploring further with respect of policy interventions, environmental communication and messaging. Corresponding with the three clusters’ pattern differences in ACCRs, respondent segments also differ in age, sex, income, education, and political affiliation. Further research is needed to shed light on the social characteristics and context of people who transitioned in one direction compared to the other.

## Supplementary information

Appendices

## Data Availability

The data used in the study are available from the Understanding Society (https://www.understandingsociety.ac.uk/) by registering with and accepting the end user licence at the UK Data Service (https://ukdataservice.ac.uk/).

## Footnotes

1 This dataset can be downloaded, ordered or analysed online by registering with and accepting the end user licence at the UK Data Service (https://ukdataservice.ac.uk/).

2 The General Population sample is based on two separate samples of residential addresses in England, Scotland and Wales and in Northern Ireland. The Ethnic Minority Boost sample was designed to provide at least 1000 adults from each of five groups: Indian, Pakistani, Bangladeshi, Caribbean, and African. The British Household Panel Survey sample (BHPS) created at Wave 2 consisted of all members from the BHPS (a predecessor survey) who were still active at Wave 18 of the BHPS and who had not refused to take part in UKHLS. The Immigrant and Ethnic Minority Boost Sample was added at Wave 6 and includes people who were born outside the United Kingdom (“immigrants”) and members of five ethnic minority groups: Indian, Pakistani, Bangladeshi, Caribbean, and African. For details about each sample, see the Study Design section of the Main-survey user-guide: https://www.understandingsociety.ac.uk/documentation/mainstage/user-guides/main-survey-user-guide/.

3 There is also a household questionnaire and a youth questionnaire, which collects information about households and participants aged 10–15 years old, respectively. However, these are not our target population, so these data were not used. For details about the questionnaires, see Interview Process in the Main-survey user-guide: https://www.understandingsociety.ac.uk/documentation/mainstage/user-guides/main-survey-user-guide/.

4 We follow van der Linden’s (2017) definition of ACCRs and select five ACCRs variables because they evaluated how people perceive and are concerned about the timescale, trend, consequences, and crisis of climate change. Variables were not included in our analysis if they only captured attitudes towards climate change (as our aim is to capture how people perceive (and worry about) the risk of climate change, not actions impacting climate change and the relationship between climate change attitudes and behaviour Furthermore, statements that explicitly concerned attitudes to the general environment without referencing climate change were also excluded. Responses to such statements are indicative of participants’ opinions on their lifestyle or habits. Including such variables in our analysis would likely introduce additional variance into the analysis, conflating attitudes towards risks of climate change from other environmental issues and producing results relating to a behavioural dimension. Undoubtedly, such variables do have a relationship with climate change attitudes, but they are likely to be confounded. We did however include two items, which refer to the “environmental crisis”—our rationale here was that because of the extensive and ongoing media engagement with the topic climate change is likely to be at the forefront of respondents’ minds when considering forthcoming crises. We also considered but declined to use a sixth item, capturing people’s perception of climate change risk on a distant timescale: ‘People in the UK will be affected by climate change in the next 200 years’. We decided not to use it because it asked about timescales far beyond the predictive capacity of even our best scientific climate models, and it had a relatively low item-rest correlation (*r*_wave4_ = 0.39, *r*_wave10_ = 0.38).

5 According to the user-guide for UKHLS dataset of Understanding Society, income variables can be negative due to self-employment reported losses or those dedicated for missing values. A value of 0 means their income was 0 and negative values except for missing values are actual negative values reported. In our study, only 0.10% of the respondents in wave 4 and 0.05% of the respondents in wave 10 reported negative values for income. For more details on negative value and missing values of variables please see https://www.understandingsociety.ac.uk/documentation/mainstage/user-guides/main-survey-user-guide/missing-values.

6 5 = Degree, including first and higher degrees (e.g., MSc, Ph.D), also foundation degrees, graduate membership of a professional Institute, PGCE. 4 = Other higher education, including diplomas, teaching qualifications (excluding PGCE), and nursing or other medical qualifications. 3 = A/AS Levels, Welsh and international baccalaureates, highers/advanced highers (Scotland), and certificate of sixth year studies. 2 = GCSE/O level, STANDARD/Ordinary (O) grade/Lower (Scotland), CSE, and other school qualifications. 1 = No qualification mentioned.

7 When classifying parties based on ideology in waves 4 and 10 data, we relied on the variable “LRGEN” in the 2014, 2017, and 2019 Chapel Hill Expert Survey dataset, which asked experts to rate the positions of each party in terms of its overall ideological stance, with 0 meaning extreme left, 10 meaning extreme right. We define left parties as those that score below 5 on this scale and right parties as those above 5, with a score of 5 being classed as centre. For more details on the recoding of other parties please see Supplementary Appendix B. Wave 4 data was collected from 2012 to 2014. Although this is a sort of period in the UK politics where the liberal democrat party went into a government with conservative party, the average placement of liberal democrat party scored at 4.86 at 2014. For detailed information on the political spectrum see Supplementary Appendix B and https://www.chesdata.eu/2014-chapel-hill-expert-survey, https://yougov.co.uk/topics/politics/articles-reports/2014/07/23/britains-changing-political-spectrum, https://www.chesdata.eu/2019-chapel-hill-expert-survey, https://www.chesdata.eu/2017-chapel-hill-expert-survey, https://www.belfastlive.co.uk/news/uup-needs-reclaim-ground-centre-20574823, “*The Independent*, 22 April 2019”. Archived from the original on 23 April 2019, Jarrett (2016).

8 We also used the Hausman test to examine the Independence of Irrelevant Alternatives (IIA) for the multinomial logistic regression model, excluding the Sceptical cluster and Paradoxical cluster, respectively. The Hausman test was significant at the 0.05 level when excluding the larger paradoxical cluster but was not significant when excluding the smaller sceptical cluster in two waves. In both cases the differences in estimated coefficients were very small and did not affect our conclusions.

9 We also carried out a sensitivity analysis of *k*-means clustering and multinomial logistic regression between raw data and weighted sample, which applied survey weight constructed by combining design weights and non-response weights. Results showed that three-cluster means patterns of ACCRs and odds ratios of age, sex, education, income, and political affiliation on cluster membership have not been changed. Given that waves 4 and 10 data has no over-represented socio-demographics compared to population proportions, we chose to present the results using the unweighted sample in the paper.

10 We tested for multicollinearity among predictors (sex, age, income, education, political affiliation) of cluster membership using multiple regression analysis; tolerance was 0.870 in wave 4 and 0.901 in wave 10 data, indicating no collinearity problem.

## Informed content

This article does not contain any studies with human participants performed by any of the authors.
