# Supplementary material for: Attitudes to climate change risk: classification of and transitions in the UK population between 2012 and 2020
Source: Humanit Soc Sci Commun. 2022 Aug 18;9(1):279. doi: 10.1057/s41599-022-01287-1 (PMC9386649; doi:10.1057/s41599-022-01287-1)
Supplement: Supplementary file 1 — Appendices [file 41599_2022_1287_MOESM1_ESM.docx]

**Appendix A. Missing data analysis of waves 4 and 10 data**

[**1. Missing data analysis plan** 1](#_Toc99643417)

[**2.** **Summary of missing data analysis** 2](#_Toc99643418)

[**3.** **Wave 4 data** 2](#_Toc99643419)

[3.1 Univariate missingness for each variable 2](#_Toc99643420)

[3.2 Multivariate missing data pattern 3](#_Toc99643421)

[3.2.1 Missing reasons of climate change questions in pattern 34 3](#_Toc99643422)

[3.2.2 Missing reasons of climate change questions in pattern 4 4](#_Toc99643423)

[3.3 Logistic regression analysis for each individual variable for each pattern 6](#_Toc99643424)

[3.3.1 Descriptive analysis of demographics in three missing data pattern. 6](#_Toc99643425)

[3.3.2 People with pattern 34 (missing by refusal or don’t know) Versus. People with no pattern 34 6](#_Toc99643426)

[3.3.3 People with pattern 34 (missing by refusal or don’t know) Versus. People with pattern 1 7](#_Toc99643427)

[3.3.4 People with pattern 4 Versus. People with no pattern 4 7](#_Toc99643428)

[3.3.5 People with pattern 4 Versus. People with pattern 1. 8](#_Toc99643429)

[**4** **Wave 10 data** 9](#_Toc99643430)

[4.1 Univariate missingness for each variable 9](#_Toc99643431)

[4.2 Multivariate missing data pattern 9](#_Toc99643432)

[4.2.1 Missing reasons of climate change in pattern 39 10](#_Toc99643433)

[4.2.2 Missing reasons of climate change questions in pattern 7 10](#_Toc99643434)

[4.3 Logistic regression analysis for each individual variable for each pattern 12](#_Toc99643435)

[4.3.1 Descriptive analysis of demographics in three missing data pattern. 12](#_Toc99643436)

[4.3.2 People with pattern 5 Versus. People with no pattern 5 12](#_Toc99643437)

[4.3.3 People with pattern 5 Versus. People with pattern 1 13](#_Toc99643438)

[4.3.4 People with pattern 7 Versus. People with no pattern 7. 13](#_Toc99643439)

[4.3.5 People with pattern 7 Versus. People with pattern 1. 14](#_Toc99643440)

[4.3.6 People with pattern 39 (missing by refusal or don’t know) Versus. People with no pattern 39. 14](#_Toc99643441)

[4.3.7 People with pattern 39 (missing by refusal or don’t know) Versus. People with pattern 1. 15](#_Toc99643442)

[**5** **Summary table of missing data analysis with waves 4 and 10 data** 15](#_Toc99643443)

# Missing data analysis plan

To give a description of the types of missingness and evaluate the plausibility of the Missing At Completely Random (MACR), Missing At Random (MAR), or Missing Not At Random (MNAR), a univariate missing pattern was firstly used to indicate the situation where missing data occur only in each climate change risk attitudes variable and each demographic variable. Secondly, we utilized multivariate missing pattern to display missing data for a set of all above variables and to look at the different patterns of missingness. Thirdly, we created a dummy variable to indicate what patterns of missing data for each individual participant, especially those who have got most missingness. Then, we used logistic regression model to evaluate if there is any relationship between any of the variable and other variables in the mode and the missingness for that outcome.

1. **Summary of missing data analysis**

Univariate missingness of wave 4 data indicated that the percentage of missing data among five climate change risk attitudes variables range from 17.81% to 18.75%, in which around 93% to 97% are due to proxy respondents or not applicable and 0.2% were missing by error or implausible responses (see Tables 1 and 2 for more details). The percentage of missing data in the five climate change risk attitudes variables in wave 10 data range from 6.84% to 7.76%, in which proxy and inapplicable and missing by error or implausible accounted for 78.6% to 89.2% (see Tables 11 and 12). Therefore, both waves 4 and 10 data showed that only a very few of missing values are recorded as refusal or don’t know and warrant missingness analysis.

Multivariate missing data pattern analysis of wave 4 data suggested three major types of missing data pattern, pattern 1, pattern 4, and pattern 34 (See Table 5 for more details). Pattern 1 refers to those people with fully observed data (N = 37878). Pattern 4 stands for those people with fully observed data except the “Affected within 30 years” variable (N = 417). Pattern 34 is comprised of 8210 people with none of responses of climate change questions and only demographic responses. These three patterns account for the vast majority of the differences in missing data patterns, other missing data patterns are quite sparse (in the context of large survey data with 47071 respondents).

Wave 10 data indicated four major missing data patterns, patterns 1, 5, 7, and 39. Pattern 1 refers to those people with fully observed data (N = 30981). Pattern 5 stands for those people with fully observed data except the “education” variable (N = 482). Pattern 7 is comprised of 399 people with none of “Affected within 30 years” response. Pattern 39 stands for those people with no response to any of the five climate change questions (N = 2100). In all of the patterns, sex and income are always observed. These four patterns account for the vast majority of the differences in missing data patterns, other missing data patterns are quite sparse in the context of large survey with 34318 respondents (see Table 14 for more details). It is presumably missing data of other patterns are rounding errors, which are quite trivial and much less likely to be causing our study trouble.

Missingness was well predicted by demographic variables (sex, age, income, education, and political affiliation) used as predictors in the multinomial regression models with two waves data, making the MAR missingness assumption plausible for the analysis (see section 3 and 4 for more details of missingness type diagnosis in two waves data).

1. **Wave 4 data**
   1. **Univariate missingness for each variable**

As shown in Table 1, the univariate missingness suggested that sex, income and age has no missingness. The percent of missing data among five climate change risk attitudes variables range from 17.81% to 18.75%. In wave 4 data, around 93% to 97% of the missing values of each climate change question are due to proxy (N = 3935) and inapplicable (N = 4255), only .2% (N = 19) were missing by error or implausible (see Table 2).

Table 1. Univariate missingness for each variable in wave 4.

| **Variable** | **Missing** | **Total** | **Percent Missing (%)** |
| --- | --- | --- | --- |
| Major Disaster | 8,483 | 47,071 | 18.02 |
| Crisis Exaggerated | 8,450 | 47,071 | 17.95 |
| Beyond Control | 8,446 | 47,071 | 17.94 |
| Too Far in Future | 8,383 | 47,071 | 17.81 |
| Affected within 30 years | 8,828 | 47,071 | 18.75 |
| Sex | 0 | 47,071 | 0 |
| Education | 219 | 47,071 | .47 |
| Political affiliation | 42 | 47,071 | .09 |
| Income | 0 | 47,071 | 0 |
| Age | 0 | 47,071 | 0 |

Note: AACR cluster, attitudes toward climate change cluster.

Table 2. Value label of climate change risk attitudes questions for wave 4 data.

|  | Value label | Major Disaster | Beyond Control | Crisis Exaggerated | Too Far in Future | Affected within 30 years |
| --- | --- | --- | --- | --- | --- | --- |
|  |  | Freq. | Freq. | Freq. | Freq. | Freq. |
| Missing values | **Missing** | **19** | **19** | **19** | **19** | **19** |
|  | **Inapplicable** | **4,255** | **4,255** | **4,255** | **4,255** | **4,255** |
|  | **Proxy** | **3,935** | **3,935** | **3,935** | **3,935** | **3,935** |
|  | Refusal | 33 | 34 | 35 | 33 | 45 |
|  | Don't know | 241 | 203 | 206 | 141 | 574 |
| Valid response | Strongly agree | 4,104 | 1,467 | 2,712 | 1,787 | 29,851 (Yes, I believe) |
|  | Tend to agree | 12,645 | 5,503 | 9,684 | 6,038 | 8,392 (No, I don't believe) |
|  | Neither agree nor disagree | 14,497 | 14,221 | 15,149 | 13,250 | - |
|  | Tend to disagree | 5,486 | 13,256 | 8,117 | 12,151 | - |
|  | Strongly disagree | 1,856 | 4,178 | 2,959 | 5,462 | - |
|  | Total | 47,071 | 47,071 | 47,071 | 47,071 | 47,071 |

Note: “Missing”, missing by error or implausible”. “Inapplicable”, this information is missing because the person was never asked this question as they were not eligible for it. “Proxy”, sometimes when a person cannot participate in the interview, someone else in the household (generally their spouse or partner or adult children) answers questions on their behalf, that is, by proxy. This questionnaire is a much shorter questionnaire asking factual information. So, if a question was not included in the proxy questionnaire and the person gave a proxy interview, this variable will be missing for them. “Refused”, when the respondent refuses to answer. “Don't know”, when the respondent does not know the answer.

- 1. **Multivariate missing data pattern**

As shown in Table 5, there are three major types of missing data pattern in this dataset, pattern 1, pattern 4, and pattern 34. Pattern 1 refers to those people with fully observed data (N = 37878), pattern 4 stands for those people with fully observed data except the “Affected within 30 years” variable (N = 417). Pattern 34 is comprised of 8210 people with none of responses of climate change questions and only demographic responses. In all of the patterns, age, sex, and income are always observed. These three patterns account for the vast majority of the differences in missing data patterns, other missing data patterns are quite sparse in the context of large survey data with 47071 respondents. It is presumably showed that missing data of other patterns are rounding errors, which are quite trivial and much less likely to be causing our study trouble.

### 3.2.1 Missing reasons of climate change questions in pattern 34

Among 8210 people who are in missing data pattern 34 (full missingness of five climate change questions), 4227 were missing by inapplicable, 3865 were missing by proxy, 19 were missing by error or implausible, only 99 were missing by refusal or having no ideas (see Table 3). Based on the wave 4 questionnaire, only people who selected computer assisted personal interviews (CAPI) and had agreed to self-completion or where the sample month is from 12^th^ to 24^th^ and mode is telephone have been asked climate change modules. Therefore, 4227 people were not eligible for climate change questions, 3865 people were represented by a proxy with consequently no responses to attitude or value questions. That means, 98.6% of the missing data in pattern 34 were structurally missing. 19 people answered climate change but missed during the data coding and editing period. Only the rest (N = 99) with missingness of climate change questions were presumably MCAR or MAR and warrant further missingness analysis.

Table 3. Missing values of climate change risk attitudes questions in pattern 34.

| Missing values | Major Disaster | Beyond Control | Crisis Exaggerated | Too Far in Future | Affected within 30 years |
| --- | --- | --- | --- | --- | --- |
|  | Freq. | Freq. | Freq. | Freq. | Freq. |
| **Missing** | **19** | **19** | **19** | **19** | **19** |
| **Inapplicable** | **4,227** | **4,227** | **4,227** | **4,227** | **4,227** |
| **Proxy** | **3,865** | **3,865** | **3,865** | **3,865** | **3,865** |
| Refusal | 27 | 27 | 28 | 27 | 28 |
| Don't know | 72 | 72 | 71 | 72 | 71 |
| Total | 8210 | 8210 | 8210 | 8210 | 8210 |

### 3.2.2 Missing reasons of climate change questions in pattern 4

Among 417 people who are in missing data pattern 4 (missingness of “Affected within 30 years”), 406 were missing by virtue of responding don’t know and 11 were missing by refusal (see Table 4). That means all of the missing data in pattern 4 were presumably MACR or MAR and warrant further missingness analysis.

Table 4. Missing values of climate change risk attitudes questions “Affected within 30 years” in pattern 4.

| Missing values | Affected within 30 years |
| --- | --- |
|  | Freq. |
| Missing | **-** |
| Inapplicable | **-** |
| Proxy | **-** |
| Refusal | 11 |
| Don't know | 406 |
| Total | 417 |

Table 5. Missing data pattern of climate change attitudes variables and demographic variables in wave 4 data.

| **Missing data pattern** | **1** | **2** | **3** | **4** | **5** | **6** | **7** | **8** | **9** | **10** | **11** | **12** | **13** | **14** | **15** | **16** | **17** | **18** | **19** | **20** | **21** | **22** | **23** | **24** | **25** | **26** | **27** | **28** | **29** | **30** | **31** | **32** | **33** | **34** | **35** | **36** |
| --- | --- | --- | --- | --- | --- | --- | --- | --- | --- | --- | --- | --- | --- | --- | --- | --- | --- | --- | --- | --- | --- | --- | --- | --- | --- | --- | --- | --- | --- | --- | --- | --- | --- | --- | --- | --- |
| Major Disaster | **x** | x | x | **x** | x | x | x | x | x | x | x | x | x | x | x | x | x | x | x |  |  |  |  |  |  |  |  |  |  |  |  |  |  |  |  |  |
| Crisis Exaggerated | **x** | x | x | **x** | x | x | x | x | x | x | x | x |  |  |  |  |  |  |  | x | x | x | x | x | x | x | x |  |  |  |  |  |  |  |  |  |
| Beyond Control | **x** | x | x | **x** | x | x | x | x |  |  |  |  | x | x | x |  |  |  |  | x | x | x | x |  |  |  |  | x | x | x |  |  |  |  |  |  |
| Too Far in Future | **x** | x | x | **x** | x | x |  |  | x | x |  |  | x | x |  | x | x |  |  | x | x |  |  | x | x |  |  | x | x |  | x | x |  |  |  |  |
| Affected within 30 years | **x** | x | x |  |  |  | x |  | x |  | x |  | x |  | x | x |  | x |  | x |  | x |  | x |  | x |  | x |  |  | x |  | x |  |  |  |
| Sex | **x** | x | x | **x** | x | x | x | x | x | x | x | x | x | x | x | x | x | x | x | x | x | x | x | x | x | x | x | x | x | x | x | x | x | **x** | x | x |
| Education | **x** | x |  | **x** | x |  | x | x | x | x | x | x | x | x | x | x | x | x | x | x | x | x | x | x | x | x | x | x | x | x | x | x | x | **x** | x |  |
| Political affiliation | **x** |  | x | **x** |  | x | x | x | x | x | x | x | x | x | x | x | x | x | x | x | x | x | x | x | x | x | x | x | x | x | x | x | x | **x** |  | x |
| Income | **x** | x | x | **x** | x | x | x | x | x | x | x | x | x | x | x | x | x | x | x | x | x | x | x | x | x | x | x | x | x | x | x | x | x | **x** | x | x |
| Age | **x** | x | x | **x** | x | x | x | x | x | x | x | x | x | x | x | x | x | x | x | x | x | x | x | x | x | x | x | x | x | x | x | x | x | **x** | x | x |
| N | **37878** | 38 | 121 | **417** | 1 | 2 | 12 | 6 | 27 | 4 | 4 | 12 | 29 | 10 | 1 | 9 | 4 | 6 | 7 | 55 | 18 | 1 | 3 | 13 | 4 | 2 | 3 | 19 | 11 | 3 | 14 | 14 | 14 | **8210** | 3 | 96 |

## 3.3 Logistic regression analysis for each individual variable for each pattern

To evaluate whether people with different patterns of missingness systematically different from one another, we specified logistic regression models to examine the relationship between the three major patterns of missingness and outcome variables.

### 3.3.1 Descriptive analysis of demographics in three missing data pattern.

Table 6 shows a description of the demographics in terms of mean, minimum and maximum. We intend to examine how do people who have responses to climate change questions missing differ from those who do not. Compared to patterns 1 and 34, pattern 4 is more likely to be the older people and those on a lower income. While pattern 34 is more inclined to be those people who have GCSE etc educational attainment and support other party compared to patterns 1 and 4.

Table 6. Descriptive analysis of demographics in three missing data patterns in wave 4 data.

|  | | **Age** | **Income** | **Sex** | **Education** | **Political affiliation** |
| --- | --- | --- | --- | --- | --- | --- |
| Mean | Pattern 1 | 47.13 | 1667.83 | .44 | 3.06 | 2.86 |
|  | Pattern 4 | 62.74 | 1173.81 | .45 | 2.23 | 2.83 |
|  | Pattern 34 | 55.77 | 1263.81 | .34 | 2.14 | 3.12 |
| Minimum | Pattern 1 | 16 | 0 | 0 | 1 | 1 |
|  | Pattern 4 | 16 | 0 | 0 | 1 | 1 |
|  | Pattern 34 | 16 | 0 | 0 | 1 | 1 |
| Maximum | Pattern 1 | 100 | 27472.7 | 1 | 5 | 4 |
|  | Pattern 4 | 98 | 9341.79 | 1 | 5 | 4 |
|  | Pattern 34 | 95 | 8499.67 | 1 | 5 | 4 |
| N | Pattern 1 | 37878 | 37878 | 37878 | 37878 | 37878 |
|  | Pattern 4 | 417 | 417 | 417 | 417 | 417 |
|  | Pattern 34 (missing by refusal or don’t know) | 99 | 99 | 99 | 99 | 99 |

Note. Sex: 0 = female, 1 = male. Education: 1 = No qualification, 2 = GCSE etc, 3 = A-level etc, 4 = Other higher education, 5 = Degree. Political affiliation: 1 = right-wing, 2 = left-wing, 3 = other party supporter, 4 = None/Inapplicable/other.

### 3.3.2 People with pattern 34 (missing by refusal or don’t know) Versus. People with no pattern 34

Table 7 showed the difference in demographics between people who have got missingness of climate change due to refusal or don’t know and people who have not got all of climate change questions missing. Results indicated that the missingness of climate change questions is related to age and education, implying people who have got missingness of climate change questions by refusal and don’t know were plausibly MAR.

Table 7. Logistic regression of missingness of climate change variables and demographic variables between people with pattern 34 and people with no pattern 34.

| **Pattern 34 (missing by refusal or don’t know)** | **Coef.** | **Std. Err.** | **z** | **P>z** | **95% CI** | |
| --- | --- | --- | --- | --- | --- | --- |
| Age | .01367 | .01 | 2.28 | .023 | .00 | .03 |
| Sex |  |  |  |  |  |  |
| Male | -.36609 | .22 | -1.68 | .093 | -.79 | .06 |
| Income | -.00005 | .00 | -.47 | .637 | .00 | .00 |
| Education |  |  |  |  |  |  |
| GCSE etc | -.82378 | .26 | -3.22 | .001 | -1.33 | -.32 |
| A-level etc | -1.47030 | .38 | -3.87 | .000 | -2.21 | -.73 |
| Other higher education | -1.46683 | .45 | -3.23 | .001 | -2.36 | -.58 |
| Degree | -1.44467 | .39 | -3.73 | .000 | -2.20 | -.68 |
| Political affiliation |  |  |  |  |  |  |
| Left | .56032 | .37 | 1.51 | .131 | -.17 | 1.29 |
| Other party | -.08384 | 1.05 | -.08 | .936 | -2.14 | 1.98 |
| None/Inapplicable/other | .61313 | .35 | 1.75 | .081 | -.07 | 1.30 |
| _cons | -6.28353 | .57 | -1.97 | 0 | -7.41 | -5.16 |
| N | 46810 | | | | | |

### 3.3.3 People with pattern 34 (missing by refusal or don’t know) Versus. People with pattern 1

Table 8 shows the difference in demographics between people who have no climate change responses due to refusal and don’t know and people who have fully answered questions. Results indicated that the missingness of climate change questions is related to age, other higher education, and political affiliation, implying missingness of climate change by refusal or don’t know in pattern 34 was plausibly MAR, compared to people who have got no responses missing.

Table 8. Logistic regression of missingness of climate change variables and demographic variables between people with pattern 34 and people with pattern 1.

| **Pattern 34 (missing by refusal or don’t know)** | **Coef.** | **Std. Err.** | **z** | **P>z** | **95% CI** | |
| --- | --- | --- | --- | --- | --- | --- |
| Age | .0161 | .01 | 2.59 | .010 | .00 | .03 |
| Sex | -.0001 | .00 | -.58 | .559 | .00 | .00 |
| Male |  |  |  |  |  |  |
| Income | -.2638 | .22 | -1.21 | .227 | -.69 | .16 |
| Education |  |  |  |  |  |  |
| GCSE etc | .5903 | .37 | 1.59 | .112 | -.14 | 1.32 |
| A-level etc | -.1089 | 1.05 | -.1 | .918 | -2.17 | 1.95 |
| Other higher education | .8326 | .35 | 2.37 | .018 | .14 | 1.52 |
| Degree |  |  |  |  |  |  |
| Political affiliation | -.9867 | .26 | -3.85 | .000 | -1.49 | -.48 |
| Left | -1.6434 | .38 | -4.33 | .000 | -2.39 | -.90 |
| Other party | -1.6564 | .45 | -3.66 | .000 | -2.54 | -.77 |
| None/Inapplicable/other | -1.6116 | .39 | -4.16 | .000 | -2.37 | -.85 |
| _cons | -6.1955 | .58 | -1.65 | .000 | -7.34 | -5.06 |
| N | 37977 | | | | | |

### 3.3.4 People with pattern 4 Versus. People with no pattern 4

Table 9 shows the difference in demographics between people who have no response to “Affected within 30 years” and those that do. Results indicated that the missingness of “Affected within 30 years” is related to age, sex, income, educational attainment higher than GCSE etc but not political affiliation, implying pattern 4 was plausibly MAR.

Table 9. Logistic regression of missingness of climate change variables and demographic variables between people with pattern 4 and people with no pattern 4.

| **Pattern 4** | **Coef.** | **Std. Err.** | **z** | **P>z** | **95% CI** | |
| --- | --- | --- | --- | --- | --- | --- |
| Age | .0393 | .00 | 12.51 | .000 | .033 | .045 |
| Sex |  |  |  |  |  |  |
| Male | -.4758 | .11 | -4.32 | .000 | -.692 | -.260 |
| Income | -.0002 | .00 | -3.23 | .001 | .000 | .000 |
| Education |  |  |  |  |  |  |
| GCSE etc | -.2212 | .12 | -1.78 | .075 | -.464 | .022 |
| A-level etc | -.7070 | .18 | -3.84 | .000 | -1.067 | -.346 |
| Other higher education | -.7848 | .21 | -3.77 | .000 | -1.193 | -.376 |
| Degree | -.6543 | .19 | -3.47 | .001 | -1.024 | -.285 |
| Political affiliation |  |  |  |  |  |  |
| Left | -.0030 | .15 | -.02 | .984 | -.291 | .285 |
| Other party | -.1868 | .40 | -.47 | .639 | -.966 | .593 |
| None/Inapplicable/other | .0384 | .14 | .28 | .777 | -.227 | .304 |
| _cons | -6.0740 | .29 | -21.3 | .000 | -6.633 | -5.515 |
| N | 46810 | | | | | |

### 3.3.5 People with pattern 4 Versus. People with pattern 1.

Table 10 shows the difference in demographics between people who have no response to “Affected within 30 years” and those that have responded. Results indicated that the missingness of “Affected within 30 years” is related to age, sex, income, education but not political affiliation, implying pattern 4 was plausibly MAR, compared to people who have got none of any questions missing.

Table 10. Logistic regression of missingness of climate change variables and demographic variables between people with pattern 4 and people with pattern 1.

| **Pattern 4** | **Coef.** | **Std. Err.** | **z** | **P>z** | **95% CI** | |
| --- | --- | --- | --- | --- | --- | --- |
| Age | .0433 | .00 | 13.15 | .000 | .037 | .050 |
| Sex | -.0002 | .00 | -3.39 | .001 | .000 | .000 |
| Male |  |  |  |  |  |  |
| Income | -.4060 | .11 | -3.66 | .000 | -.623 | -.189 |
| Education |  |  |  |  |  |  |
| GCSE etc | -.3600 | .12 | -2.89 | .004 | -.604 | -.116 |
| A-level etc | -.8504 | .18 | -4.62 | .000 | -1.211 | -.490 |
| Other higher education | -.9432 | .21 | -4.53 | .000 | -1.351 | -.535 |
| Degree | -.7940 | .19 | -4.22 | .000 | -1.163 | -.425 |
| Political affiliation |  |  |  |  |  |  |
| Left | .0266 | .15 | .18 | .857 | -.262 | .316 |
| Other party | -.1821 | .40 | -.46 | .648 | -.963 | .599 |
| None/Inapplicable/other | .2657 | .14 | 1.95 | .051 | -.001 | .532 |
| _cons | -6.1010 | .29 | -2.79 | .000 | -6.676 | -5.526 |
| N | 38295 | | | | | |

1. **Wave 10 data**
   1. **Univariate missingness for each variable**

As shown in Table 11, the univariate missingness suggested that sex and income has no missingness. The percent of missing data among five climate change risk attitudes variables range from 6.84% to 7.76%.

Table 11. Univariate missingness for each variable.

| **Variable** | **Missing** | **Total** | **Percent Missing (%)** |
| --- | --- | --- | --- |
| Major Disaster | 2,348 | 34,318 | 6.84 |
| Crisis Exaggerated | 2,355 | 34,318 | 6.86 |
| Beyond Control | 2,353 | 34,318 | 6.86 |
| Too Far in Future | 2,345 | 34,318 | 6.83 |
| Affected within 30 years | 2,662 | 34,318 | 7.76 |
| Sex | 0 | 34,318 | 0 |
| Education | 625 | 34,318 | 1.82 |
| Political affiliation | 24 | 34,318 | .07 |
| Income | 1 | 34,318 | .003 |
| Age | 12 | 34,318 | .03 |

Note: AACR cluster, attitudes toward climate change cluster.

- 1. **Multivariate missing data pattern**

As shown in Table 15, there are four major types of missing data pattern in wave 10 data, patterns 1, 5, 7, and 39. Pattern 1 refers to those people with fully observed data (N = 30981). Pattern 5 stands for those people with fully observed data except the “education” variable (N = 482). Pattern 7 is comprised of 399 people with none of “Affected within 30 years” response. Pattern 39 refers to those people with none of responses of five climate change questions (N = 2100). In all of the patterns, sex and income are always observed. These four patterns account for the vast majority of the differences in missing data patterns, other missing data patterns are quite sparse in the context of large survey with 34318 respondents (see Table 15). In wave 10 data, around 75% to 85% of the missing values are due to proxy (N = 804) and inapplicable (N = 1181, see Table 12).

Table 12. Value label of climate change risk attitudes questions for wave 10 data.

|  | Responses | Major Disaster | Beyond Control | Crisis Exaggerated | Too Far in Future | Affected within 30 years |
| --- | --- | --- | --- | --- | --- | --- |
|  |  | Freq. | Freq. | Freq. | Freq. | Freq. |
| Missing values | **Missing** | 107 | 107 | 107 | 107 | 109 |
|  | **Inapplicable** | **1,181** | **1,181** | **1,181** | **1,181** | **1,181** |
|  | **Proxy** | **804** | **804** | **804** | **804** | **804** |
|  | Refusal | 134 | 131 | 134 | 133 | 118 |
|  | Don't know | 122 | 130 | 129 | 120 | 450 |
| Valid response | Strongly agree | 5,865 | 741 | 1,111 | 871 | 26,639 (Yes, I believe) |
|  | Tend to agree | 12,969 | 3,740 | 4,920 | 3,613 | 5,017 (No, I don't believe) |
|  | Neither agree nor disagree | 9,586 | 10,420 | 10,998 | 9,267 | - |
|  | Tend to disagree | 2,788 | 12,530 | 9,399 | 10,974 | - |
|  | Strongly disagree | 762 | 4,534 | 5,535 | 7,248 | - |
|  | Total | 34,318 | 34,318 | 34,318 | 34,318 | 34,318 |

Note: “Missing”, missing by error or implausible”. “Inapplicable”, this information is missing because the person was never asked this question as they were not eligible for it. “Proxy”, sometimes when a person cannot participate in the interview, someone else in the household (generally their spouse or partner or adult children) answers questions on their behalf, that is, by proxy. This questionnaire is a much shorter questionnaire asking factual information. So, if a question was not included in the proxy questionnaire and the person gave a proxy interview, this variable will be missing for them. “Refused”, when the respondent refuses to answer. “Don't know”, when the respondent does not know the answer.

### 4.2.1 Missing reasons of climate change in pattern 39

Among 2100 people who are in missing data pattern 39 (full missingness of climate change), 1112 were missing by inapplicable, 770 were missing by proxy, 101 were missing by error, only 117 were missing by refusal or having no ideas (see Table 13). Based on wave 10 questionnaire universe, only people who selected face-to-face interview and has agreed to self-completion or mode is telephone or web have been asked climate change modules. Therefore, 1112 people were not eligible for climate change questions, 770 people selected proxy questionnaire with no attitudes or value questions. That means, 94.4% of the missing data in pattern 39 were structurally missing. 101 people answered climate change but missed during the data coding and editing period. Only the rest (N = 117) with missingness of climate change questions were presumably MCAR or MAR and warrant further missingness analysis.

Table 13. Missing values of climate change risk attitudes questions in pattern 39.

| Missing values | Major Disaster | Beyond Control | Crisis Exaggerated | Too Far in Future | Affected within 30 years |
| --- | --- | --- | --- | --- | --- |
|  | Freq. | Freq. | Freq. | Freq. | Freq. |
| **Missing** | **101** | **101** | **101** | **101** | **101** |
| **Inapplicable** | **1112** | **1112** | **1112** | **1112** | **1112** |
| **Proxy** | **770** | **770** | **770** | **770** | **770** |
| Refusal | 72 | 70 | 73 | 71 | 65 |
| Don't know | 45 | 47 | 44 | 46 | 52 |
| Total | 2100 | 2100 | 2100 | 2100 | 2100 |

### 4.2.2 Missing reasons of climate change questions in pattern 7

Among 399 people who are in missing data pattern 7 (missingness of “Affected within 30 years”), 360 were missing by having no ideas, 37 were missing by refusal and 2 were missing by error or implausible (see Table 14). That means all of the missing data in pattern 7 were presumably MACR or MAR and warrant further missingness analysis.

Table 14. Missing values of climate change risk attitudes questions in pattern 7.

| Missing values | Affected within 30 years |
| --- | --- |
|  | Freq. |
| Missing | 2 |
| Inapplicable | - |
| Proxy | - |
| Refusal | 37 |
| Don't know | 360 |
| Total | 399 |

Table 15. Missing data pattern of climate change attitudes variables and demographic variables for wave 10 data.

| **Missing data pattern** | **1** | **2** | **3** | **4** | **5** | **6** | **7** | **8** | **9** | **10** | **11** | **12** | **13** | **14** | **15** | **16** | **17** | **18** | **19** | **20** | **21** | **22** | **23** | **24** | **25** | **26** | **27** | **28** | **29** | **30** | **31** | **32** | **33** | **34** | **35** | **36** | **37** | **38** | **39** | **40** | **41** |
| --- | --- | --- | --- | --- | --- | --- | --- | --- | --- | --- | --- | --- | --- | --- | --- | --- | --- | --- | --- | --- | --- | --- | --- | --- | --- | --- | --- | --- | --- | --- | --- | --- | --- | --- | --- | --- | --- | --- | --- | --- | --- |
| Major Disaster | x | x | x | x | x | x | x | x | x | x | x | x | x | x | x | x | x | x | x | x | x |  |  |  |  |  |  |  |  |  |  |  |  |  |  |  |  |  |  |  |  |
| Crisis Exaggerated | x | x | x | x | x | x | x | x | x | x | x | x | x |  |  |  |  |  |  |  |  | x | x | x | x | x | x | x |  |  |  |  |  |  |  |  |  |  |  |  |  |
| Beyond Control | x | x | x | x | x | x | x | x | x | x | x |  |  | x | x | x | x |  |  |  |  | x | x | x | x |  |  |  | x | x | x | x | x |  |  |  |  |  |  |  |  |
| Too Far in Future | x | x | x | x | x | x | x | x |  |  |  | x |  | x | x |  |  | x | x |  |  | x | x | x |  | x |  |  | x | x | x |  |  | x | x |  |  |  |  |  |  |
| Affected within 30 years | x | x | x | x | x | x |  |  | x | x |  | x | x | x |  | x |  | x |  | x |  | x | x |  | x | x | x |  | x | x |  | x |  | x |  | x | x | x |  |  |  |
| Sex | x | x | x | x | x | x | x | x | x | x | x | x | x | x | x | x | x | x | x | x | x | x | x | x | x | x | x | x | x | x | x | x | x | x | x | x | x | x | x | x | x |
| Education | x | x | x | x |  |  | x |  | x |  | x | x | x | x | x | x | x | x | x | x | x | x |  | x | x | x | x | x | x |  | x | x | x | x | x | x | x |  | x |  |  |
| Political affiliation | x | x | x |  | x | x | x | x | x | x | x | x | x | x | x | x | x | x | x | x | x | x | x | x | x | x | x | x | x | x | x | x | x | x | x | x |  | x | x | x | x |
| Income | x |  | x | x | x | x | x | x | x | x | x | x | x | x | x | x | x | x | x | x | x | x | x | x | x | x | x | x | x | x | x | x | x | x | x | x | x | x | x | x | x |
| Age | x | x |  | x | x |  | x | x | x | x | x | x | x | x | x | x | x | x | x | x | x | x | x | x | x | x | x | x | x | x | x | x | x | x | x | x | x | x | x | x |  |
| N | **30981** | 1 | 10 | 23 | **482** | 1 | **399** | 14 | 6 | 1 | 3 | 15 | 4 | 9 | 1 | 2 | 1 | 3 | 4 | 4 | 6 | 12 | 1 | 1 | 3 | 1 | 2 | 3 | 7 | 1 | 1 | 2 | 3 | 3 | 3 | 79 | 1 | 2 | **2100** | 122 | 1 |

Note: Major missing patterns are bold.

## 4.3 Logistic regression analysis for each individual variable for each pattern

### 4.3.1 Descriptive analysis of demographics in three missing data pattern.

Table 16 displayed description of demographics in terms of mean, minimum and maximum. Compared to patterns 1 and 5, pattern 7 is more likely to be people who are at retired age, female, and GCSE etc educational attainment, while pattern 39 is more likely to be male and people who have lower income.

Table 16. Descriptive analysis of demographics in three missing data patterns.

|  |  | **Age** | **Income** | **Sex** | **Education** | **Political affiliation** |
| --- | --- | --- | --- | --- | --- | --- |
| Mean | Pattern 1 | 49.65 | 1907.83 | .44 | 3.25 | 3.70 |
|  | Pattern 5 | 48.05 | 1418.70 | .44 | - | 3.55 |
|  | Pattern 7 | 54.08 | 1443.91 | .37 | 2.57 | 3.76 |
|  | Pattern 39 | 45.50 | 1230.76 | .52 | 2.85 | 3.91 |
| Minimum | Pattern 1 | 16 | 0 | 0 | 1 | 1 |
|  | Pattern 5 | 16 | 0 | 0 | - | 1 |
|  | Pattern 7 | 16 | 0 | 0 | 1 | 1 |
|  | Pattern 39 | 16 | 0 | 0 | 1 | 1 |
| Maximum | Pattern 1 | 103 | 26634.33 | 1 | 5 | 4 |
|  | Pattern 5 | 92 | 17232.67 | 1 | - | 4 |
|  | Pattern 7 | 96 | 8333 | 1 | 5 | 4 |
|  | Pattern 39 | 96 | 6158.43 | 1 | 5 | 4 |
| N | Pattern 1 | 30981 | 30981 | 30981 | 30981 | 30981 |
|  | Pattern 5 | 482 | 482 | 482 | - | 482 |
|  | Pattern 7 | 399 | 399 | 399 | 399 | 399 |
|  | Pattern 39 (missing by refusal or don’t know) | 117 | 117 | 117 | 117 | 117 |

Note. Sex: 0 = female, 1 = male. Education: 1 = No qualification, 2 = GCSE etc, 3 = A-level etc, 4 = Other higher education, 5 = Degree. Political affiliation: 1 = right-wing, 2 = left-wing, 3 = other party supporter, 4 = None/Inapplicable/other.

### 4.3.2 People with pattern 5 Versus. People with no pattern 5

We examined the difference in responses of demographics between people who have no education responses (pattern 5) and people who have not got education missing (no pattern 5). Results indicated that the missingness of education question is more likely to be those people with lower income, left-winger and other party supporter, implying pattern 5 was plausibly MAR (see Table 17).

Table 17. Logistic regression of people with pattern 5 and people with no pattern 5.

| **Pattern 5** | **Coef.** | **Std. Err.** | **z** | **P>z** | **95% CI** | |
| --- | --- | --- | --- | --- | --- | --- |
| Age | -.0027 | .002 | -1.14 | .255 | -.007 | .002 |
| Sex |  |  |  |  |  |  |
| Male | .1007 | .094 | 1.07 | .283 | -.083 | .285 |
| Income | -.0002 | .000 | -6.03 | .000 | .000 | .000 |
| Political affiliation |  |  |  |  |  |  |
| Left | .8900 | .259 | 3.43 | .001 | .382 | 1.398 |
| Other party | 2.0550 | .786 | 2.62 | .009 | .515 | 3.595 |
| None/Inapplicable/other | -.0882 | .238 | -.37 | .711 | -.555 | .378 |
| _cons | -3.8165 | .275 | -13.88 | .000 | -4.355 | -3.278 |
| N | 34282 | | | | | |

### 4.3.3 People with pattern 5 Versus. People with pattern 1

Table 18 showed the difference in demographics between people who have no political affiliation responses (pattern 5) and people who have fully answered questions (pattern 1). Results indicated that the missingness of education question is related to income and political affiliation, implying pattern 5 was plausibly MAR, compared to people who have got none of any questions missing.

Table 18. Logistic regression of people with pattern 5 with people with pattern 1.

| **Pattern 5** | **Coef.** | **Std. Err.** | **z** | **P>z** | **95% CI** | |
| --- | --- | --- | --- | --- | --- | --- |
| Age | -.0020 | .002 | -.81 | .416 | -.007 | .003 |
| Sex |  |  |  |  |  |  |
| Male | .1281 | .094 | 1.36 | .173 | -.056 | .312 |
| Income | -.0003 | .000 | -6.47 | .000 | .000 | .000 |
| Political affiliation |  |  |  |  |  |  |
| Left | .8896 | .259 | 3.43 | .001 | .381 | 1.398 |
| Other party | 2.0301 | .786 | 2.58 | .010 | .490 | 3.571 |
| None/Inapplicable/other | -.0017 | .238 | -.01 | .994 | -.468 | .465 |
| _cons | -3.8197 | .275 | -13.87 | .000 | -4.359 | -3.280 |
| N | 31464 | | | | | |

### 4.3.4 People with pattern 7 Versus. People with no pattern 7.

Table 19 showed the difference in demographics between people who have no “Affected within 30 years” response and people who have not got this question missing. Results indicated that the missingness of this question is related to sex, age, income, and education, implying pattern 7 was plausibly MAR.

Table 19. Logistic regression of missingness of “Affected within 30 years” question and demographic variables between people with pattern 7 and people with no pattern 7.

| **Pattern 7** | **Coef.** | **Std. Err.** | **z** | **P>z** | **95% CI** | |
| --- | --- | --- | --- | --- | --- | --- |
| Age | .0063 | .003 | 2.27 | .023 | .001 | .012 |
| Sex |  |  |  |  |  |  |
| Male | -.2474 | .107 | -2.30 | .021 | -.458 | -.037 |
| Income | -.0001 | .000 | -2.19 | .029 | .000 | .000 |
| Education |  |  |  |  |  |  |
| GCSE etc | -.3753 | .144 | -2.60 | .009 | -.658 | -.093 |
| A-level etc | -.6795 | .174 | -3.92 | .000 | -1.020 | -.339 |
| Other higher education | -.5758 | .188 | -3.07 | .002 | -.944 | -.208 |
| Degree | -1.3526 | .203 | -6.67 | .000 | -1.750 | -.955 |
| Political affiliation |  |  |  |  |  |  |
| Left | -.4582 | .334 | -1.37 | .170 | -1.113 | .197 |
| Other party | .0000 | (empty) |  |  |  |  |
| None/Inapplicable/other | -.0879 | .233 | -.38 | .706 | -.545 | .370 |
| _cons | -3.7911 | .326 | -11.62 | .000 | -4.430 | -3.152 |
| N | 33641 | | | | | |

### 4.3.5 People with pattern 7 Versus. People with pattern 1.

Table 20 showed the difference in demographics between people who have no “Affected within 30 years” response and people who have not got this question missing. Results indicated that the missingness of this question is related to sex, age, income, and education, implying pattern 7 was plausibly MAR, compared to people who have got none of any questions missing.

Table 20. Logistic regression of missingness of “Affected within 30 years” question and demographic variables between people with pattern 7 and people with pattern 1.

| **Pattern 7** | **Coef.** | **Std. Err.** | **z** | **P>z** | **95% CI** | |
| --- | --- | --- | --- | --- | --- | --- |
| Age | .0066 | .003 | 2.33 | .020 | .001 | .012 |
| Sex |  |  |  |  |  |  |
| Male | -.2165 | .108 | -2.01 | .044 | -.427 | -.006 |
| Income | -.0001 | .000 | -2.4 | .016 | .000 | .000 |
| Education |  |  |  |  |  |  |
| GCSE etc | -.4726 | .145 | -3.26 | .001 | -.756 | -.189 |
| A-level etc | -.7877 | .174 | -4.52 | .000 | -1.129 | -.446 |
| Other higher education | -.6902 | .188 | -3.67 | .000 | -1.059 | -.321 |
| Degree | -1.4766 | .203 | -7.27 | .000 | -1.875 | -1.079 |
| Political affiliation |  |  |  |  |  |  |
| Left | -.4537 | .334 | -1.36 | .175 | -1.109 | .201 |
| Other party | .0000 | (empty) |  |  |  |  |
| None/Inapplicable/other | .0003 | .233 | 0 | .999 | -.457 | .458 |
| _cons | -3.7052 | .328 | -11.3 | .000 | -4.348 | -3.063 |
| N | 31363 | | | | | |

### 4.3.6 People with pattern 39 (missing by refusal or don’t know) Versus. People with no pattern 39.

Table 21 showed the difference in demographics between people who have no climate change and people who have not got these questions missing. Results indicated that the missingness of climate change questions is related to sex, age, income, and education, implying pattern 39 was plausibly MAR.

Table 21. Logistic regression of missingness of climate change variables and demographic variables between people with pattern 39 and people with no pattern 39.

| **Pattern 39 (missing by refusal or don’t know)** | **Coef.** | **Std. Err.** | **z** | **P>z** | **95% CI** | |
| --- | --- | --- | --- | --- | --- | --- |
| Age | -.0118 | .005 | -2.37 | .018 | -.021 | -.002 |
| Sex |  |  |  |  |  |  |
| Male | .4748 | .188 | 2.52 | .012 | .106 | .844 |
| Income | -.0003 | .000 | -3.71 | .000 | -.001 | .000 |
| Education |  |  |  |  |  |  |
| GCSE etc | -.5331 | .281 | -1.90 | .058 | -1.084 | .018 |
| A-level etc | -.9023 | .327 | -2.76 | .006 | -1.543 | -.261 |
| Other higher education | -.7028 | .382 | -1.84 | .066 | -1.452 | .046 |
| Degree | -.5390 | .319 | -1.69 | .091 | -1.165 | .087 |
| Political affiliation |  |  |  |  |  |  |
| Left | .7301 | 1.121 | .65 | .515 | -1.467 | 2.927 |
| Other party | .0000 | (empty) |  |  |  |  |
| None/Inapplicable/other | 1.5172 | 1.006 | 1.51 | .132 | -.455 | 3.490 |
| _cons | -5.6943 | 1.078 | -5.28 | .000 | -7.807 | -3.582 |
| N | 33641 | | | | | |

### 4.3.7 People with pattern 39 (missing by refusal or don’t know) Versus. People with pattern 1.

Table 22 showed the difference in demographics between people who have no climate change response and people who have got none of questions missing. Results indicated that the missingness of climate change is related to sex, income, and education, implying pattern 39 was plausibly MAR, compared to people who have got none of any questions missing.

Table 22. Logistic regression of missingness of climate change variables and demographic variables between people with pattern 39 and people with pattern 1.

| **Pattern 39 (missing by refusal or don’t know)** | **Coef.** | **Std. Err.** | **z** | **P>z** | **95% CI** | |
| --- | --- | --- | --- | --- | --- | --- |
| Age | -.0117 | .005 | -2.35 | .019 | -.021 | -.002 |
| Sex |  |  |  |  |  |  |
| Male | .4717 | .188 | 2.50 | .012 | .103 | .841 |
| Income | -.0003 | .000 | -3.74 | .000 | -.001 | .000 |
| Education |  |  |  |  |  |  |
| GCSE etc | -.5460 | .281 | -1.94 | .052 | -1.097 | .005 |
| A-level etc | -.9169 | .327 | -2.80 | .005 | -1.558 | -.276 |
| Other higher education | -.7190 | .382 | -1.88 | .060 | -1.468 | .030 |
| Degree | -.5606 | .319 | -1.76 | .079 | -1.187 | .065 |
| Political affiliation |  |  |  |  |  |  |
| Left | .7257 | 1.121 | .65 | .517 | -1.471 | 2.922 |
| Other party | .0000 | (empty) |  |  |  |  |
| None/Inapplicable/other | 1.5207 | 1.006 | 1.51 | .131 | -.452 | 3.493 |
| _cons | -5.6625 | 1.077 | -5.26 | .000 | -7.774 | -3.551 |
| N | 33064 | | | | | |

1. **Summary table of missing data analysis with waves 4 and 10 data**

Table 23. Missing data pattern and missingness type for wave 4 data.

| Major missing data pattern | N | Missing variables | M_age_ | M_sex_ | M_income_ | M_education_ | M_political affiliation_ | Evaluation | Missingness type |
| --- | --- | --- | --- | --- | --- | --- | --- | --- | --- |
| 1 | 37878 | None | 47.13 | 1667.83 | 0.44 | 3.06 | 2.86 | - | None |
|  |  |  |  |  |  |  |  | - | None |
| 4 | 417 | Affected within 30 years | 62.74 | 1173.81 | 0.45 | 2.23 | 2.83 | P4 vs. everyone | MAR |
|  |  |  |  |  |  |  |  | P4 vs. P1 | MAR |
| 34 (missing by refusal or don’t know) | 99 | Five climate change questions | 55.77 | 1263.81 | 0.34 | 2.14 | 3.12 | P34 vs. everyone | MAR |
|  |  |  |  |  |  |  |  | P34 vs. P1 | MAR |

Table 24. Missing data pattern and missingness type for wave 10 data.

| Major missing data pattern | N | Missing variables | M_age_ | M_sex_ | M_income_ | M_education_ | M_political affiliation_ | Evaluation | Missingness type |
| --- | --- | --- | --- | --- | --- | --- | --- | --- | --- |
| 1 | 30981 | None | 49.65 | 1907.83 | 0.44 | 3.25 | 3.7 | - | None |
|  |  |  |  |  |  |  |  | - | None |
| 5 | 482 | Education | 48.05 | 1418.7 | 0.44 | - | 3.55 | P5 vs. everyone | MAR |
|  |  |  |  |  |  |  |  | P5 vs. P1 | MAR |
| 7 | 399 | Affected within 30 years | 54.08 | 1443.91 | 0.37 | 2.57 | 3.76 | P7 vs. everyone | MAR |
|  |  |  |  |  |  |  |  | P7 vs. P1 | MAR |
| 39 (missing by refusal or don’t know) | 117 | Climate change | 45.5 | 123.76 | 0.52 | 2.85 | 3.91 | P39 vs. everyone | MAR |
|  |  |  |  |  |  |  |  | P39 vs. P1 | MAR |

**Appendix B.**

| Party id | Party name | Obs | Mean | SD | Min | Max | Political position |
| --- | --- | --- | --- | --- | --- | --- | --- |
| 1101 | Conservative Party | 17 | 7.12 | .70 | 6 | 9 | Right |
| 1102 | Labour Party | 17 | 1.94 | .83 | 1 | 4 | Left |
| 1104 | Liberal Democratic Party | 17 | 4.24 | .90 | 3 | 6 | Left |
| 1105 | Scottish National Party | 16 | 3.50 | .82 | 2 | 5 | Left |
| 1106 | Plaid Cymru, Party of Wales | 11 | 3.09 | .83 | 2 | 4 | Left |
| 1107 | Green Party | 17 | 2.00 | .87 | 0 | 3 | Left |
| 1108 | United Kingdom Independence Party | 16 | 8.69 | .70 | 8 | 10 | Right |
| 1110 | Brexit Party | 15 | 8.20 | .68 | 7 | 10 | Right |

Table B. Overall ideological stance of the party in 2019 Chapel Hill expert survey.

Sources: 2019 Chapel Hill expert survey. https://www.chesdata.eu/2019-chapel-hill-expert-survey

**Recode missing data of political affiliation**

In our study, we selected the question “which political party do you feel closest to” in political and social engagement module to measure people’s political affiliation. Based on wave 4 and wave 10 questionnaire universe, people who are eligible to be asked political party are those respondents whose interview mode is face-to-face and have agreed to self-completion or interview mode of telephone or web, as well as support a particular political party or closer to one party than the others. Because of that, more than half of the respondents were missing by inapplicable (46.63%), proxy (8.36%), refusal (.3%), or don’t know (.21%) in wave 4 data (see Table C below). Although k-means cluster analysis of climate change risk attitudes is independent of any demographics, large missing data of political affiliation leads to a low valid sample for analysing cluster membership of climate change risk attitudes by demographics. Therefore, we recoded missing data into a new category of political affiliation, None/Inapplicable/Other, representing those people who respond none of those political party. The situation is the same with wave 10 data, nearly 90% of the respondents were missing by inapplicable (28.03%), missing (57.44%), proxy (2.34%), refused (.26%), or don’t know (.06%, see Table C). Consistently, we recoded those missing data into a new category of political affiliation, None/Inapplicable/Other. These responses are not coded as missing data, but as valid responses showing the respondent had no particular affiliation with a political party. This category played no role in the cluster membership by political affiliation and has been removed in the presentation. The only effect of this category is to include those people who have fully answered climate change questions, and other demographic information including education, income, age, and sex.

Table C. Response value and frequency of political affiliation in waves 4 and 10 data.

|  | **Value label** | **Wave 4** | | | **Wave 10** | | |
| --- | --- | --- | --- | --- | --- | --- | --- |
|  |  | **Value** | **Absolute frequency** | **Relative frequency** | **Value** | **Absolute frequency** | **Relative frequency** |
| Missing values | Missing | - | - | - | -9 | 19711 | 57.44% |
|  | Inapplicable | -8 | 21949 | 46.63% | -8 | 9619 | 28.03% |
|  | Proxy | -7 | 3935 | 8.36% | -7 | 804 | 2.34% |
|  | Refused | -2 | 143 | .30% | -2 | 89 | .26% |
|  | Don’t know | -1 | 100 | .21% | -1 | 19 | .06% |
| Valid response | Conservatives | 1 | 6757 | 14.35% | 1 | 1396 | 4.07% |
|  | Labour | 2 | 9177 | 19.50% | 2 | 1721 | 5.01% |
|  | Liberal Democrat | 3 | 1643 | 3.49% | 3 | 468 | 1.36% |
|  | Scottish National Party | 4 | 600 | 1.27% | 4 | 86 | .25% |
|  | Plaid Cymru | 5 | 155 | .33% | 5 | 8 | .02% |
|  | Green Party | 6 | 457 | .97% | 6 | 203 | .59% |
|  | Ulster Unionist | 7 | 352 | .75% | 7 | 1 | .00% |
|  | Social Democratic and Labour Party | 8 | 237 | .50% | - | - | - |
|  | Alliance Party | 9 | 125 | .27% | 9 | 2 | .01% |
|  | Democratic unionist | 10 | 374 | .79% | - | - | - |
|  | Sinn fein | 11 | 227 | .48% | - | - | - |
|  | UK Independence Party | - | - | - | 12 | 27 | .08% |
|  | The Brexit Party | - | - | - | 14 | 120 | .35% |
|  | Change UK - The Independent Group | - | - | - | 15 | 5 | .01% |
|  | Can't vote | 96 | 42 | .09% | 96 | 24 | .07% |
|  | Other party | 97 | 798 | 1.70% | 97 | 15 | .04% |
| Total |  |  | 47071 | 10.00% |  | 34318 | 10.00% |

Note: -, empty or out of recording.
